# Is the Ne operation of the helium ion microscope suitable for electron backscatter diffraction sample preparation?

**DOI:** 10.3762/bjnano.12.73

**Published:** 2021-08-31

**Authors:** Annalena Wolff

**Affiliations:** 1 Central Analytical Research Facility, Institute for Future Environments, Queensland University of Technology, 2 George St, Brisbane 4000, QLD, Australia

**Keywords:** electron backscatter diffraction (EBSD), Ga, helium ion microscope (HIM), ion polishing, Ne

## Abstract

Electron backscatter diffraction (EBSD) is a powerful characterization technique which allows the study of microstructure, grain size, and orientation as well as strain of a crystallographic sample. In addition, the technique can be used for phase analysis. A mirror-flat sample surface is required for this analysis technique and different polishing approaches have been used over the years. A commonly used approach is the focused ion beam (FIB) polishing. Unfortunately, artefacts that can be easily induced by Ga FIB polishing approaches are seldom published. This work aims to provide a better understanding of the underlying causes for artefact formation and to assess if the helium ion microscope is better suited to achieve the required mirror-flat sample surface when operating the ion source with Ne instead of He. Copper was chosen as a test material and polished using Ga and Ne ions with different ion energies as well as incident angles. The results show that crystal structure alterations and, in some instances, phase transformation of Cu to Cu_3_Ga occurred when polishing with Ga ions. Polishing with high-energy Ne ions at a glancing angle maintains the crystal structure and significantly improves indexing in EBSD measurements. By milling down to a depth equaling the depth of the interaction volume, a steady-state condition of ion impurity concentration and number of induced defects is reached. The EBSD measurements and Monte Carlo simulations indicate that when this steady-state condition is reached more quickly, which can be achieved using high-energy Ne ions at a glancing incidence, then the overall damage to the specimen is reduced.

## Introduction

**Figure 1:**
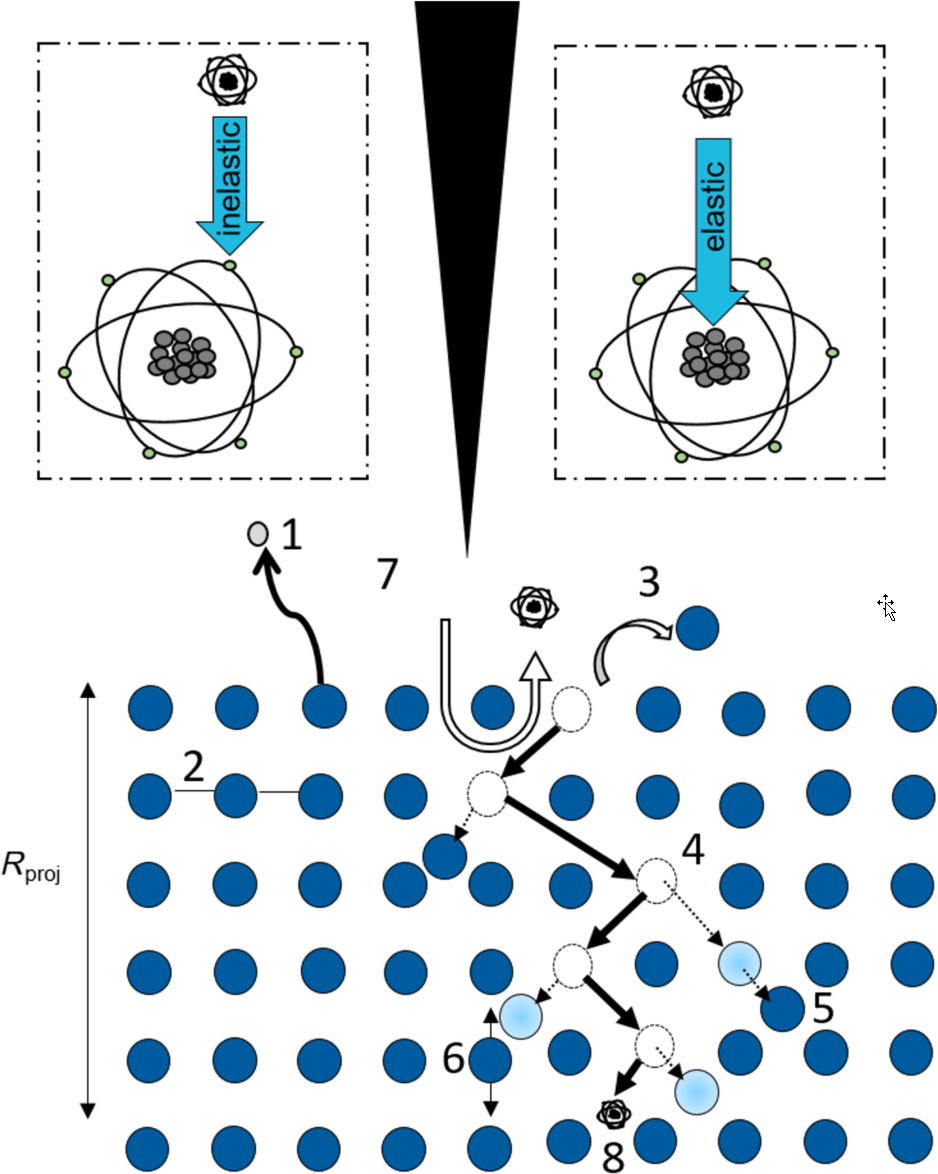
Illustration of the underlying ion–solid interactions that occur when irradiating a sample with different ion species. The incident ions (black) can interact via inelastic (top left inset) or elastic collisions (top right inset) with the sample atoms (blue). As a result of the inelastic interactions, secondary electrons (1) can be generated or polymerization (2) can occur. The elastic interactions can lead to sputtering (3), vacancy formation (4), displacements (5), phonons (6), backscattered ions (7), and ion implantation (8). The number of the different interactions created are given for different ion species when considering 10,000 incident ions impacting on a silicon sample. These numbers were obtained from SRIM simulations.

The helium ion microscope (HIM) has sparked interest in many disciplines since its commercial release in the first decade of the 21st century [1]. From its beginnings as primarily an imaging tool [2–9] it was established as a key tool in nanofabrication [10–15], defect engineering [16–17], and recently for material analysis [18–19]. The extended range of applications in which the second-generation HIM (Orion Nanofab) is used for nowadays is supported by using up to three different ion species (He, Ne, and Ga). The versatility in its application can be understood when considering ion–solid interactions which occur when an energetic ion interacts with a specimen. An overview of the different interaction types is illustrated in Figure 1. The ions, irrespective of the ion species, interact with the sample atoms via nuclear and electronic interactions. The electronic interactions lead to secondary electron emission and polymerization while the nuclear interactions lead to sputtering, sample atom displacements, replacement collisions, vacancy formation, and a collision cascade as well as backscattered ions, secondary ion emission, and ion implantation. A more detailed description of ion–solid interactions can be found in [20–21]. The difference between different ion species, energies, and incident angles lies in the statistics of the ion–solid interactions and determines the application space for ion species. Higher energy (25–30 keV) He ions predominantly interact via electronic interactions while Ga ions of the same energy predominantly interact via nuclear interactions, making the latter an ideal candidate for sputtering applications. Neon interacts almost equally via electronic and nuclear interactions at those energies and allows for fast material removal per incident ion. As an inert ion species, neon has been proven advantageous for processing semiconducting materials in which Ga induces sample alterations and material behavior changes due to doping [22]. Not all nuclear interactions lead to sputtering. If the sample atom cannot be removed from the sample because of insufficient energy transfer or because the sample atom cannot exit the sample due to its sub-surface position, defects such as interstitials or vacancies can be created [23]. This can induce a significant amount of crystal structure alterations in a sample and thus artefacts. While these artefacts are well recognized for transmission electron microscopy (TEM) lamella preparation, in which the lamella preparation steps are designed to reduce the thickness of the amorphous layer on each side of the TEM lamella and to minimize artefacts [24], procedures and induced artefacts for other techniques such as electron backscatter diffraction (EBSD) polishing are not well documented in the literature.

The work presented here looks at the ion-beam-induced artefacts when polishing copper for EBSD measurements for different ion species (Ga, Ne) as well as polishing protocols. EBSD is a characterization technique in scanning electron microscopes (SEMs) that allows for the study of microstructure, local texture, grain size and orientations, as well as the strain of crystalline samples [25]. A detailed overview of this technique can be found in [26]. EBSD can be used for phase identification, which makes it a powerful complementary technique to energy-dispersive X-ray spectroscopy (EDS) and wavelength dispersive spectroscopy (WDS). The sample surface is conventionally tilted to 70° for the measurement and the backscatter diffraction of the SEM beam electrons leads to the formation of Kikuchi patterns on the EBSD phosphor screen which are characteristic of the crystal structure and orientation of the sample at the scan point. EBSD is a surface-sensitive technique and the signal comes from approximately the first 20 nm of the sample [27]. A thorough sample preparation and mirror-finished polish without inducing a significant number of artefacts in the sample top layer is, therefore, required for this technique.

Numerous sample preparation procedures exist to achieve the required mirror finish of the sample surface. Depending on the material, mechanical polishing [28], electropolishing [29], or ion polishing [30] are the key techniques. A thorough overview of the different EBSD preparation techniques can be found elsewhere [31]. Throughout the past years, Ga-focused ion beam/scanning electron microscopes (Ga FIB/SEMs) have been used to polish samples [32–33]. Although it is recognized within the FIB community that Ga can induce artefacts in the sample [34–37], many of the encountered artefacts, which can potentially lead to misinterpretation of the results, are unfortunately never published. Besides the initial study by Michael [34], which showed that Ga ion polishing can phase transform Cu to Cu_3_Ga as well as a follow up study investigating microtextural modifications in samples when using a Ga FIB [35], a recent study reported transformations of a crystal structure from hcp to fcc when polishing Ti with a FIB [38]. The work presented here looks at ion–solid interactions and aims to provide a better understanding of FIB polishing-induced artefacts. The work aims to assess if Ne is a more suitable ion species than Ga for EBSD sample preparation. Cu has been previously reported to phase transform under Ga FIB polishing and conventional Cu TEM grids are, therefore, used here as a test material and are polished using Ga and Ne ions of different energies, incident angles, and ion doses. Both normal and glancing angles are investigated, the former for completeness and to allow comparison to literature and the later since it is the conventionally used geometry for polishing. Changes in the crystal structure, which would lead to altered EBSD results, are assessed by EBSD measurements as well as by scanning transmission electron microscopy (STEM), TEM, TEM selected area electron diffraction (SAED), and TEM dark-field (DF) measurements. The results for each experiment are compared to those of a non-irradiated area of the Cu TEM grid. Monte Carlo simulations of the occurring ion–solid interactions are evaluated to determine the resulting interaction volume of the ions for the various conditions as well as vacancy formation and implanted impurity concentration. In addition, the achieved results are compared to those achieved by the conventionally used argon ion polishing (PIPS) and electropolishing approaches.

## Results and Discussion

**Figure 2:**
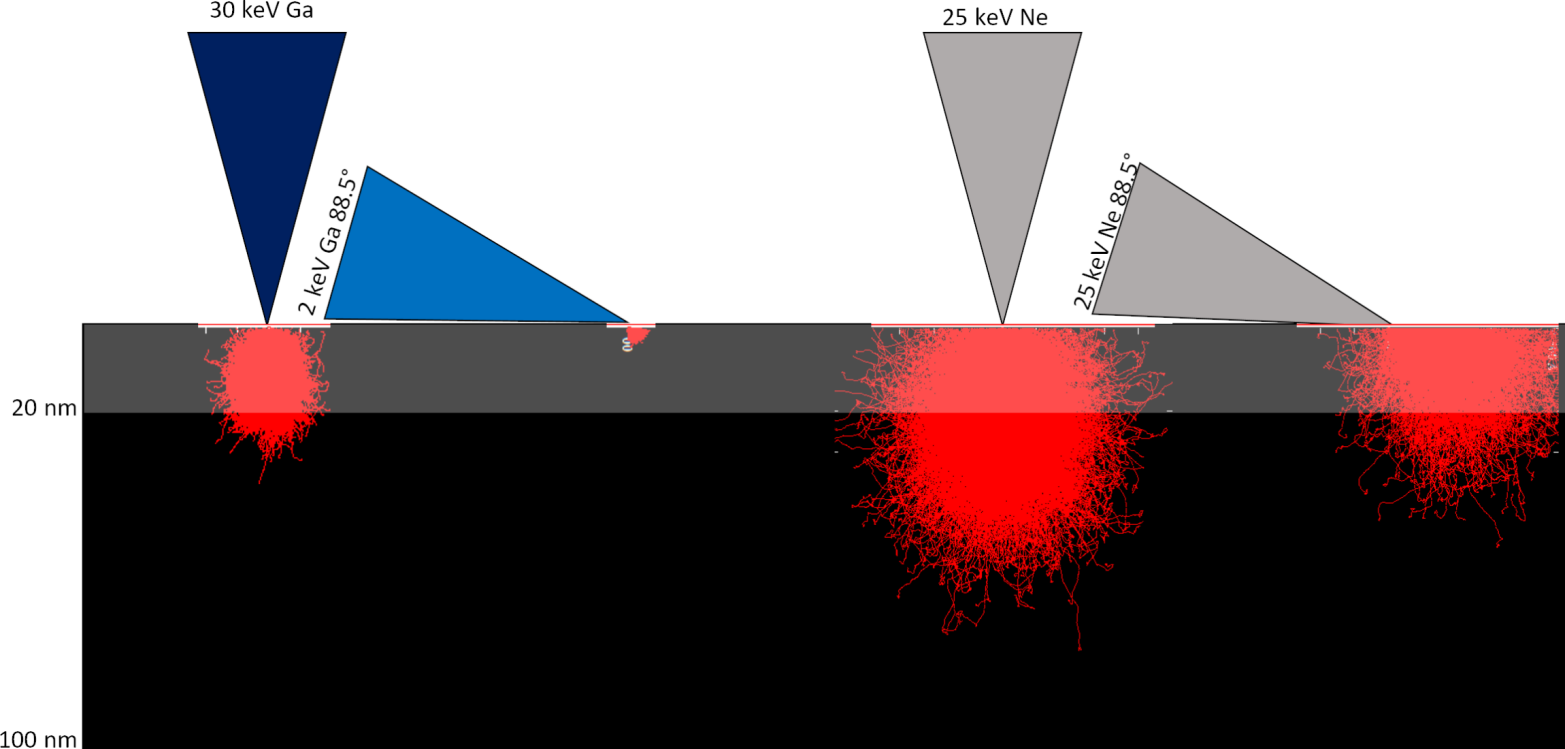
Illustration of the experimental polishing setup for the different ion species, ion energies, and incident angles. The experiments with Ga ions are displayed in blue on the left. The experiments using Ne ions are displayed in grey on the right. The EBSD signal depth is highlighted by the transparent rectangle. The SRIM-simulated ion trajectories for the different ion species, ion energies, and incident angles are shown in red.

To assess the impact of polishing samples for EBSD with a focused ion beam, Cu TEM grids were polished using different ion species (Ga, Ne) at different energies and incidence angles. A schematic overview of the experiments is given in Figure 2. STEM, TEM, SAED, as well as EBSD measurements were performed to assess if and how the crystal structure of the sample was altered during different polishing procedures. For each sample, EBSD measurements were performed on the bulk specimen, thus the native Cu TEM grid serves as a true control sample. Thin foils of each specimen were then prepared using FIB for the TEM measurements. For each sample, the results were evaluated and compared to an unpolished area on the Cu TEM grids (control experiments). To ensure that the FIB TEM lamella preparation does not affect the TEM measurements of the crystal structure, TEM foils were additionally prepared using PIPS and electropolishing. The measurements and comparison of the FIB-prepared TEM lamella as well as the electropolished and PIPS-prepared thin foils are given in the Supporting Information File 1 and show that the FIB preparation of the thin foils for the TEM and STEM analysis did not produce artefacts that would compromise the TEM analysis. Monte Carlo simulations were performed using the program Stopping and Range of Ions in Matter (SRIM) to better understand the underpinning ion–solid interactions for the different settings [22]. The extent of created dislocations, vacancies as well as ion impurity concentration within the smaller of either the EBSD information depth (20 nm) or interaction volume depth are evaluated and correlated to the experiments.

### Non-irradiated copper

To verify the original specimen structure, TEM electron diffraction on thin foils as well as EBSD measurements on the bulk Cu TEM grid sample were performed and the results analyzed. While the EBSD measurement of the non-irradiated copper shows the original sample crystal structure, and can be considered as a true control sample, the TEM thin foils were prepared using FIB.

**Figure 3:**
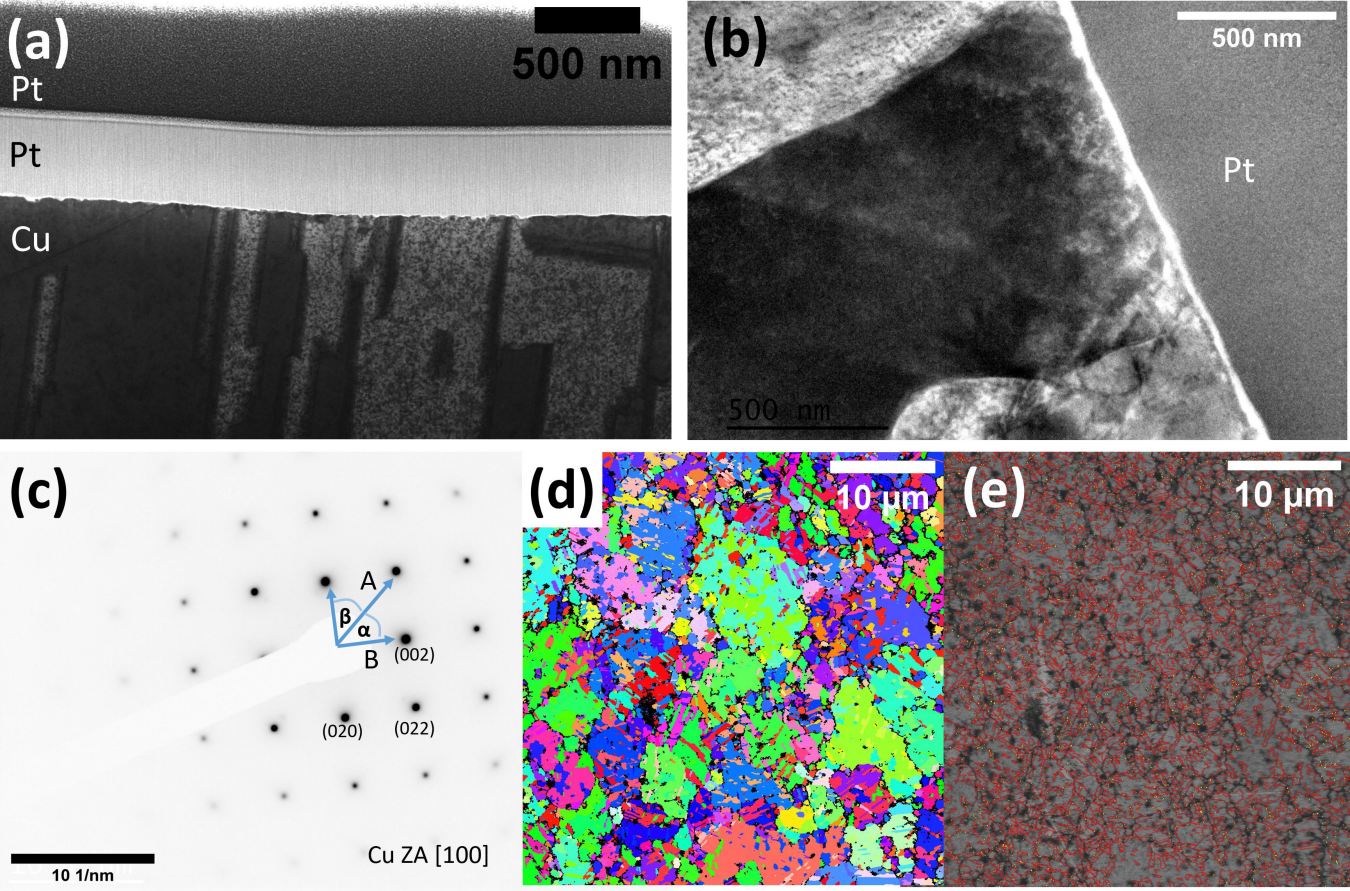
STEM, TEM, and EBSD measurements of an unpolished Cu control sample. (a) 30 kV STEM image showing a cross-sectional sample view of the Cu sample and the protective Pt-rich layers deposited with the ion beam and electron beam during TEM lamella preparation. (b) TEM image showing a cross-sectional sample view. (c) An inverted SAED pattern recorded underneath the sample surface. (d) IPF X EBSD orientation map. (e) Overlay of the band contrast and grain boundary map. HAGB are displayed in red, LAGB are displayed in yellow. The EBSD measurements were recorded on a sufficiently flat area.

The STEM (Figure 3a) measurements were taken in the Ga FIB/SEM and the TEM measurements (Figure 3b) were performed on the FIB-prepared thin TEM lamella to visualize the original grain structure on the sample surface. The corresponding SAED pattern is shown in Figure 3c. The SAED pattern was inverted for better visibility here. The angles α and β between the reflections were both measured to be 45° and the distance ratio of the reflections determined to be 1.4, suggesting a fcc crystal structure with [100] zone axis (ZA). The measured *d*-spacing for *d*_022,A_ = 0.133 nm is in agreement with the reported *d*-spacing for Cu with *d*_022_ = 0.1278 nm according to the International Centre for Diffraction Data (ICDD, PDF number 00-004-0836). The measured *d*-spacing for *d*_002,B_ = 0.192 nm is slightly larger than the reported value of *d*_002,Cu_ = 0.181 nm for Cu; however, within an acceptable discrepancy. It is likely that the stamping process used to create TEM grids has induced this strain and the result is not unexpected.

An EBSD orientation map of the non-irradiated copper sample was recorded over a 39 µm field of view using a 39 nm step size on a sufficiently flat sample area for 14.5 h. 20% zero solutions we returned for that specific area. Most areas on the sample returned up to 80% zero solutions under the same conditions due to the roughness of the surface sample (see below Figure 7). The inverse pole figure (IPF X) is shown in Figure 3d. The band contrast and grain boundary map overlay is shown in Figure 3e. A statistical evaluation of the grain boundaries shows that ≈8% of the grain boundaries are low-angle grain boundaries (LAGB). These are displayed in yellow in Figure 3e. High-angle grain boundaries (HAGB) which are displayed in red in Figure 3e correspond to 92% of the grain boundaries. These values serve as reference values throughout the work against which the ion-irradiation-induced effects are evaluated. All values can be found in Table 1.

**Table 1:** Summary of the results for different polishing experiments. The impurity concentration, sputtering yield (SY), Cu atom displacements, phase, high-angle grain boundaries and low-angle grain boundaries as well as the defect percentage determined from SRIM simulations, TEM, and EBSD measurements are given for different polishing experiments.

Polishing experiment	Impurity concentration	SY	Cu atom displacements	Phase	HAGB %	LAGB %

30 keV Ga 2247 and 3371 ions/nm^2^	12–36%	10	138	Cu_3_Ga	56	44
2 keV Ga 2247 ions/nm^2^, glancing angle	100%	5	22	Cu_3_Ga	83	17
25 keV Ne 2247 and 3371 ions/nm^2^	100%	4	180–266	Cu	33	64
25 keV Ne 2247 ions/nm^2^ glancing angle	12%	12	17	Cu	92	8
control experiment bulk non-irradiated	NA	NA	NA	Cu	92	8
10 s electropolished Cu	NA	NA	NA	Cu	61	39
PIPS	NA	—	NA	Cu	87	13

### Non-irradiated copper: electropolishing and Ar ion polishing

**Figure 4:**
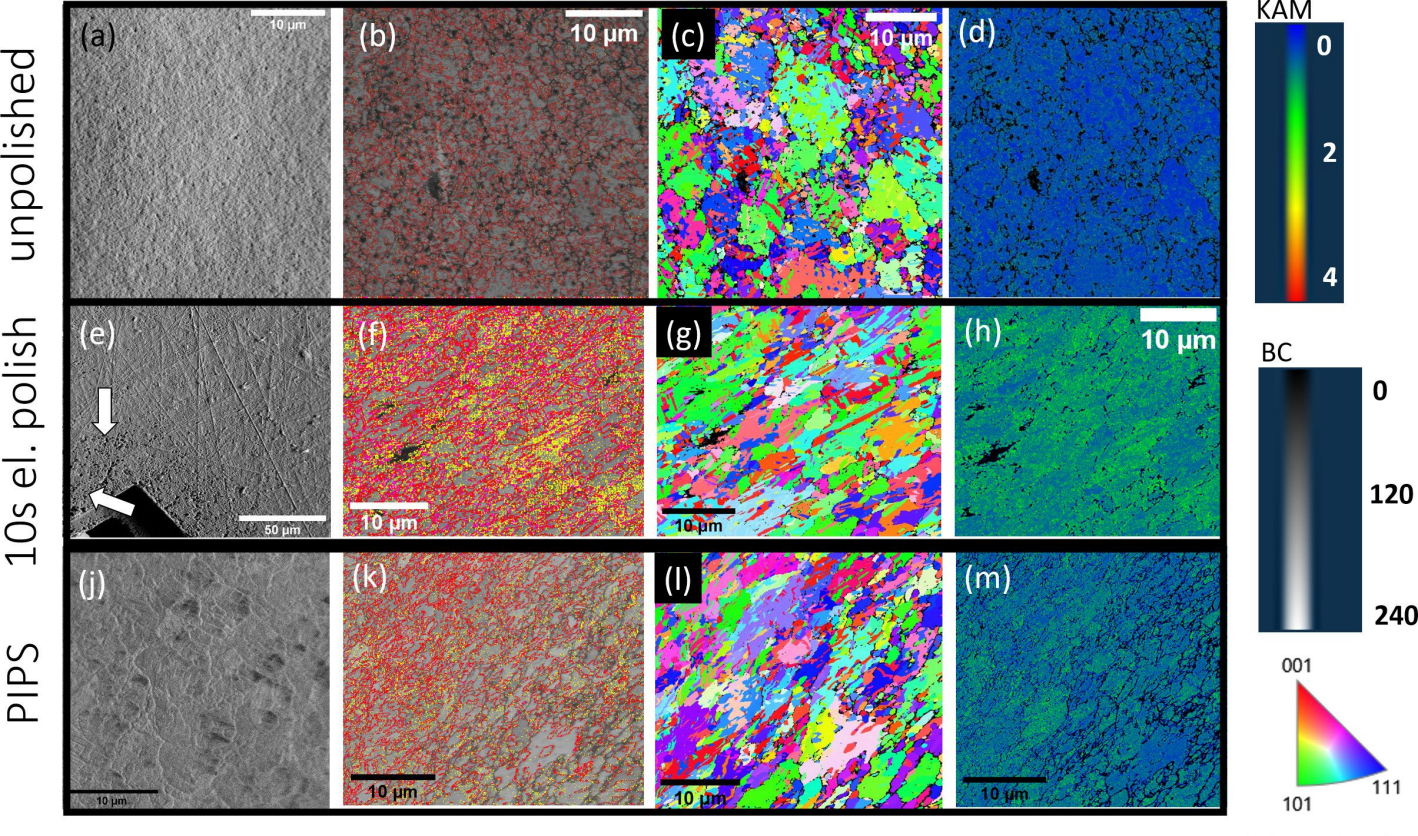
EBSD measurements of unpolished, electropolished, and low-energy Ar ion-polished Cu sample. Top row: sufficiently smooth area on an unpolished Cu sample. (a) FSD image showing the sample surface. (b) Overlay of the band contrast and grain boundary map. HAGB are displayed in red, LAGB are displayed in yellow. (c) IPF X EBSD orientation map. (d) KAM map. Blue indicates a lower kernel average misorientation, green corresponds to a higher kernel average misorientation. Middle row: 10 s electropolished Cu sample. (e) FSD image showing the sample surface. Surface alterations such as voids are highlighted by white arrows. (f) Overlay of the band contrast and grain boundary map. HAGB are displayed in red, LAGB are displayed in yellow. (g) IPF X EBSD orientation map. (h) KAM map. Blue indicates a lower kernel average misorientation, green corresponds to a higher kernel average misorientation. Bottom row: PIPS polished Cu sample. (j) FSD image showing the sample surface. (k) Overlay of the band contrast and grain boundary map. HAGB are displayed in red, LAGB are displayed in yellow. (l) IPF X EBSD orientation map. (m) KAM map. Blue indicates a lower kernel average misorientation, green corresponds to higher kernel average misorientation.

To evaluate the effect of electropolishing and argon ion polishing on EBSD measurements, the Cu sample was polished using these conventional techniques. The measurements as well as the corresponding measurements for the unpolished control sample are given in Figure 4 to enable a direct comparison. While a significant reduction in zero solutions (4%) and thus a well-indexed EBSD map (Figure 4g) could be achieved by electropolishing the sample for 10 s, a higher number of LAGB (67%) were found in comparison to the control sample (Figure 4f). In addition, the kernel average misorientation (KAM) map, (shown in Figure 4h) shows a higher level of misorientation compared to the control sample. This suggests that the used electropolishing protocol induced some artefacts in this sample which could lead to misinterpretation for stress/strain analysis of the sample. The forward scatter diffraction (FSD) image (Figure 4e) shows void formation on the sample surface and significant sample surface alterations close to the area that was polished. These surface alterations become less severe at larger distances from the central polished area as evidenced in the top right corner of the FSD image. The results suggest that electropolishing yields well-indexed EBSD maps. However, the induced changes in the crystal structure make the used electropolishing protocol not feasible for stress and strain analysis of the Cu sample.

Argon ion polishing of the Cu sample produced better results than electropolishing. The FSD image (Figure 4j) reveals grains and does not show obvious sample surface alterations in comparison to the electropolishing approaches. While the number of LAGB with 17% in the grain boundary map (Figure 4k) is still slightly larger than that for the control experiment, it is lower than the observed number for electropolishing. The level of misorientation in the KAM maps (Figure 4m) is significantly reduced in comparison to the electropolishing KAM maps. However, it is still larger than that for the original control experiment suggesting that some changes to the crystal structure were induced by this polishing approach. While a well-indexed EBSD map (Figure 4l) could be recorded, the induced crystallographic artefacts could mean a challenging stress/strain analysis.

### Irradiation of copper at 0° incidence angle

To assess the effect of ion irradiation, the copper TEM lamella grids were irradiated with Ga ions using a Ga FIB/SEM or Ne ions using HIM. An ion dose of 3371 ions/nm^2^ was chosen to allow a comparison with a previously reported study on Ga-induced phase transformations in copper [34]. A lower ion dose (2247 ions/nm^2^) was also evaluated as it corresponds to the dose that is achieved for commonly reported EBSD polishing time values over larger areas [39].

#### 30 keV Ga ion irradiation at 0° incidence angle

The ion trajectory plot (Figure 2) obtained using SRIM shows that the interaction volume depth of the 30 keV Ga ions in Cu is ≈25 nm and is in the same range as the EBSD signal information depth of 20 nm. Throughout the interaction depth, 786 vacancies are created per incident ion while ten atoms are sputtered. The total number of induced defects and number of implanted ions is limited in FIBs that are optimized for patterning applications (such as Ga FIB/SEM or HIM when operated with Ne). The highest concentration of ion implantation and defects is reached once the sample has been milled down to a depth which corresponds to the interaction volume depth. This is referred to as the steady-state condition. The time it takes to mill the sample to that depth and until the steady-state condition is reached determines the maximal area of specific ion implantation concentration. The calculations presented here determine the upper limit since sputtered and backscattered ions are not considered here. To determine the time until this steady-state condition is reached when using 30 keV Ga ions at a frontal irradiation, the copper sample was milled for 3 min using a 0.3 nA beam current (1872 ions per 1 µs) and the milled depth was measured by TEM for different grains (see Supporting Information File 2). Both the TEM image and the FSD image, the latter is recorded with the EBSD detector and highlights the surface topography, show the different milling depths for different grains. Faster milling grains were milled to a 226 nm depth. The steady-state condition is reached after 20 s for those grains. A total of 3.8 × 10^10^ Ga ions will interact with the sample in that area during this time when irradiating a 100 µm^2^ area. This is a significantly high number of implanted Ga atoms when considering that only 2.1 × 10^11^ Cu atoms are present within a volume determined by the interaction volume depth (≈25 nm) and the 100 µm^2^ size of the irradiated area, corresponding to ≈18% of impurity concentration. The number of copper atoms within the volume was calculated using the copper mass density of 8.93 × 10^3^ kg/m^3^ and its atomic mass of 63.55 g/mol. For the slower milling grain, which only milled to a depth of 110 nm during the irradiation, the steady-state condition is reached significantly later within ≈40 s, allowing for up to 7.6 × 10^10^ Ga ions to be implanted in these grains. This corresponds to a 36% of impurity concentration and would be sufficiently high to form a Cu_3_Ga phase. This value represents an average and does not take random elements of the Ga distribution into account. The simulation results suggest that phase transformations are not likely to occur homogeneously throughout the sample but rather are confined to grains with a lower sputtering rate. Both estimated concentration values are well within the published range of 1 to 50 atom % [40].

**Figure 5:**
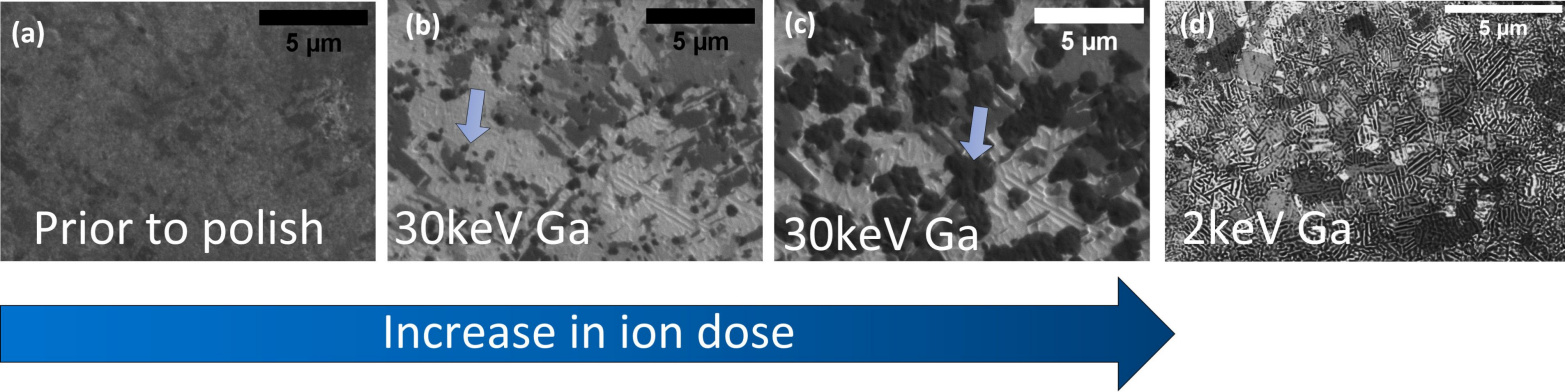
Ion channeling images showing the changes in ion channel contrast upon Ga irradiation. 30 keV Ga 0° incidence irradiation (a–c) as well as 2 keV Ga 88.5° incidence irradiation (d) are shown. (a) Start of the 30 keV Ga 0° incidence irradiation, (b) intermediate ion dose delivered, (c) irradiation dose of 3371 ions/nm^2^ delivered. (d) 2 keV Ga 88.5° incidence irradiation with a delivered irradiation dose of 2247 ions/nm^2^.

To experimentally assess the impact, the copper TEM grid was irradiated with 30 keV Ga ions at a 0° incident angle. Throughout the irradiation process changes in the ion image contrast occur with darker patches forming in the irradiated area in the ion channeling images. A detailed discussion on channeling contrast and the effect of grain orientation on milling speed can be found in [35]. The different stages of dark patch formation are shown in Figure 5a–c and a video of the occurring sample change is given in Supporting Information File 3. The patches appear to nucleate around the grain boundaries at the darker grains within the sample and then gradually increase in size and merge. The patches are highlighted with blue arrows in Figure 5b and Figure 5c. The darker grains are the slower milling grains [11] which can accumulate a higher Ga impurity concentration according to the calculations above. To verify this, EDS measurements (see Supporting Information File 4) were performed on a Ga irradiated area on slower and faster milling grains. These grains can easily be distinguished in SEM images due to their significant height differences. The Ga peak can be clearly identified in the measurements on the slower milling grain suggesting a significantly higher amount of incorporated Ga in those grains. A careful assessment of the ion channeling images and corresponding dose (see video Supporting Information File 3) suggests that significantly large dark patches are already present at an ion dose of 2247 ions/nm^2^ which is below the previously reported ion dose of 3371 ions/nm^2^ [24] (see Figure 5c). The impurity concentration for the slow milling grains is 25% when using this lower ion dose and would be sufficiently large to allow the formation of the Cu_3_Ga phase.

**Figure 6:**
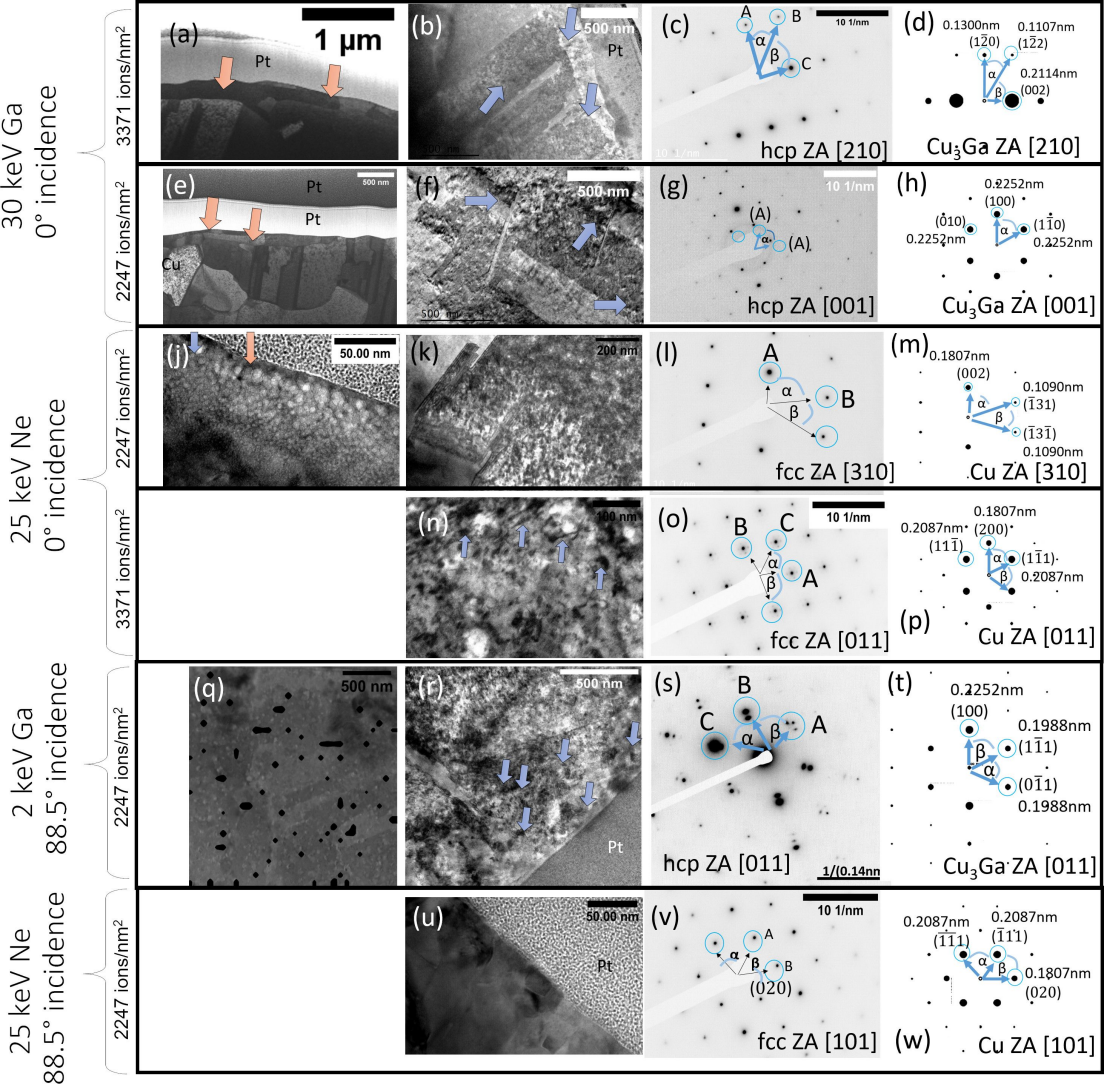
STEM, TEM, SAED, and simulated SAED patterns for the different ion irradiation approaches. (a–d) 30 keV Ga, 0° incidence irradiation, 3371 ions/nm^2^ delivered. (a) STEM showing the cross-sectional view of the irradiated sample. The orange arrows indicate the presence of a thin grain layer present on the sample surface. (b) The blue arrows in the TEM image highlight defects and precipitates. (c) A SAED pattern. (d)The corresponding SAED pattern simulation. (e–h) 30 keV Ga, 0° incidence irradiation, 2247 ions/nm^2^ delivered. (e) STEM showing the cross-sectional view of the irradiated sample. The orange arrows indicate the presence of a thin grain layer present on the sample surface. (f) The blue arrows in the TEM image highlight defects and precipitates. (g) A SAED pattern. (h) The corresponding SAED pattern simulation. (j–m) 25 keV Ne, 0° incidence irradiation, 2247 ions/nm^2^ delivered. (j) A TEM image showing Ne bubble formation underneath the sample surface within the interaction volume. (k) A TEM image showing a cross-sectional sample view. Defects and precipitates are highlighted by blue arrows. (l) A SAED pattern of the irradiated area. (m) The corresponding SAED pattern simulation. (n–p) 25 keV Ne, 0° incidence irradiation with 3371 ions/nm^2^ and 2247 ions/nm^2^ doses. (n) A TEM image showing Ne bubble formation underneath the sample surface within the interaction volume. (o) A SAED pattern of the irradiated area. (p) The corresponding SAED pattern simulation. (q–t) 2 keV Ga, 88.5° incidence irradiation, 2247 ions/nm^2^ delivered. (q) STEM DF measurement overlaid with 1σ thresholded Ga TruMap (black regions) showing the cross-sectional view. (r) TEM measurement of the irradiated area in a cross-sectional view. Defects and precipitates are highlighted by blue arrows. (s) A SAED pattern of the irradiated area. (t) The corresponding SAED pattern simulation. (u–w) 25 keV Ne, 88.5° incidence irradiation, 2247 ions/nm^2^ delivered. (u) A TEM measurement showing the cross-section of the irradiated area. (v) A SAED pattern of the irradiated area. (w) The corresponding SAED pattern simulation.

To evaluate if a significant phase transformation and sample alteration occur at a lower ion dose than previously reported, the two different ion doses (2247 ions/nm^2^ and 3371 ions/nm^2^) were assessed individually and the results compared. TEM lamellae were prepared for irradiated areas and STEM, dark-field and bright-field measurements as well as TEM SAED measurements were performed to evaluate the crystal structure underneath the irradiated area. The corresponding measurements are shown in Figure 6a–h. The SAED patters are inverted for better visibility in both cases. For the lowest ion dose of 2247 ions/nm^2^ (Figure 6e–h) the STEM measurements (Figure 6e) show a distinct layer of grains directly on the sample surface. This layer (highlighted by the blue arrows) is several hundreds of nanometers thick and significantly larger than the interaction volume. The TEM image (Figure 6f) shows a different behavior in comparison to the control sample, with more dislocations, defects, and precipitates (indicated by arrows) visible. The SAED pattern (Figure 6g) shows that the sample is crystalline. The angles between the reflections were determined to be α = 32° and β = 58°. The distance ratios between the reflections were determined to be *C*/*A* = 1.6 and *C*/*B* = 1.89 suggesting a hcp structure with [210] ZA (see Figure 6h for the simulated SAED pattern) or potentially a distorted fcc structure with 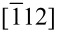 ZA. The measured *d*-spacing values of *d*_hkl,A_ = 0.135 nm, *d*_hkl,B_ = 0.114 nm, *d*_hkl,C_ = 0.215 nm match the reported distances 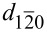 = 0.130 nm, 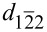 = 0.111 nm, *d*_002_= 0.211 nm for the Cu_3_Ga phase (PDF number 04-020-0557) more closely than those reported for Cu (*d*_hkl_ = 0.128 nm, *d*_hkl_ = 0.109 nm, *d*_hkl_ = 0.209 nm). For a higher ion dose of 3371 ions/nm^2^, the STEM measurements (Figure 6a) also show a layer of small grains directly underneath the irradiated surface which is distinctly different from the non-irradiated copper sample. TEM analysis (Figure 6b) of the irradiated sample shows many defects and precipitates. The defects and precipitates appear more pronounced for this higher ion dose experiment. The corresponding electron diffraction pattern is shown in Figure 6c. The angles between the reflections were determined to be α = 60°. The distance ratios between the reflections were determined to be *A*/*B* = 1 suggesting a hcp structure with [001] ZA or a fcc structure with [111] ZA. The measured *d*-spacing of *d*_hkl,A_ = 0.225 nm, matches the reported *d*-spacing values of *d*_100_ = 0.226 nm for the Cu_3_Ga phase. The simulated SAED pattern is shown in Figure 6d. It is distinctly different from those reported for Cu (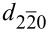 = 0.1278 nm). This is in good agreement with previous reports [34].

**Figure 7:**
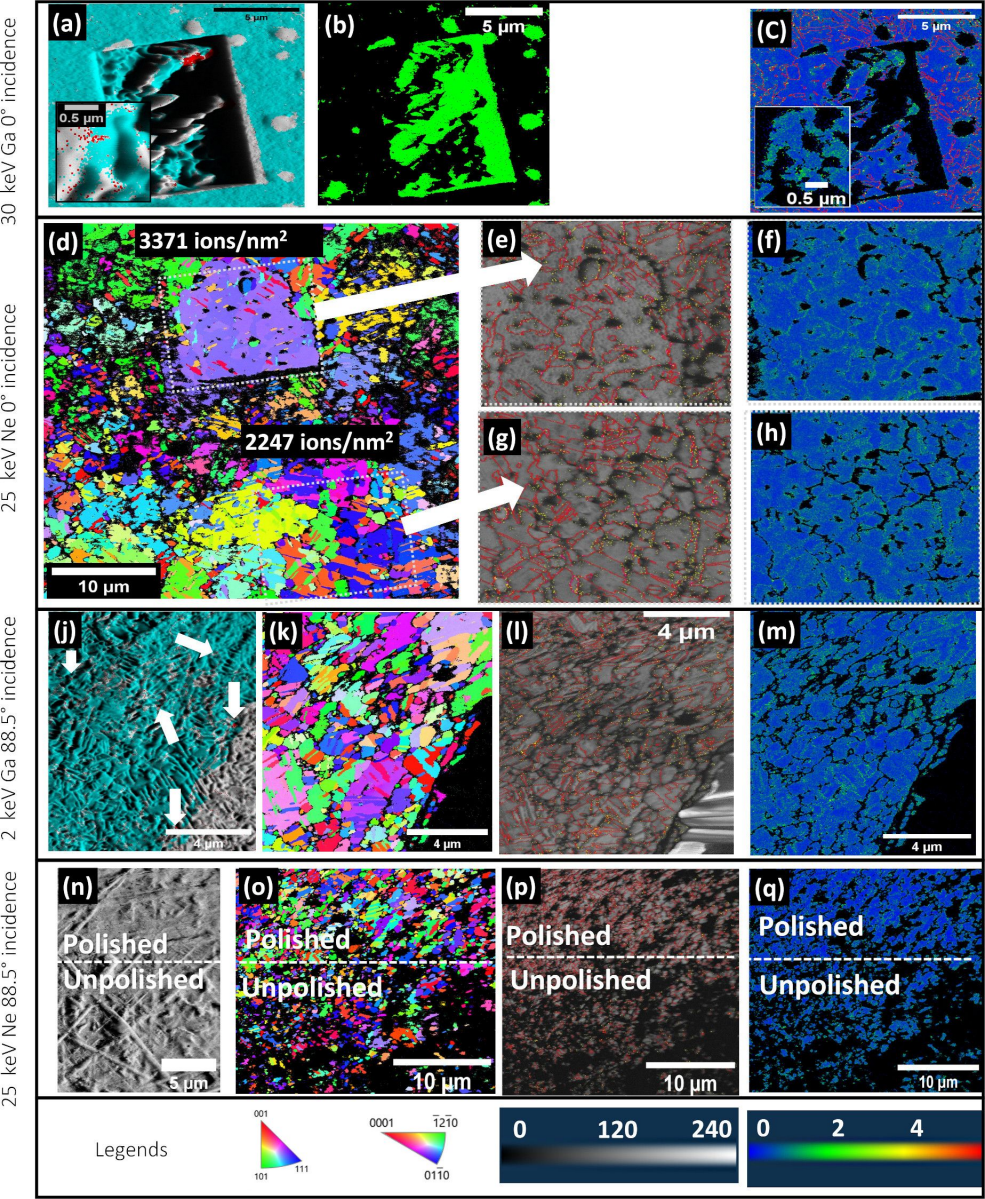
EBSD measurements and evaluation for different ion irradiation approaches. (a–c) 30 keV Ga, 0° incidence irradiation: The main images correspond to the highest ion dose of 3371 ions/nm^2^. The insets correspond to the lowest ion dose of 2247 ions/nm^2^. (a) Forward scatter diffraction image overlaid with the phase map. Blue corresponds to Cu, red corresponds to the Cu_3_Ga phase. (b) Zero solution map. The zero solutions are indicated by green points. (c) KAM map overlaid with the grain boundary map. Blue indicates a lower kernel average misorientation, green corresponds to a higher kernel average misorientation. HAGB are shown in red. LAGB are shown in yellow. (d–h) 25 keV Ne, 0° incidence irradiation with 3371 ions/nm^2^ and 2247 ions/nm^2^ doses. (d) IPF X orientation map showing two irradiated areas as well as the unpolished Cu sample surrounding the areas. (e,g) Band contrast and grain boundary maps. HAGB are displayed in red, LAGB are displayed in yellow for 3371 ions/nm^2^ (e) and 2247 ions/nm^2^ (g). (f,h) KAM maps for 3371 ions/nm^2^ (f) and 2247 ions/nm^2^ (h). A higher misorientation in both KAM maps is shown in green. (j–m) 2 keV Ga, 88.5° incidence irradiation with 2247 ions/nm^2^: EBSD and ion channeling measurements of 2 keV Ga glancing angle irradiated area with 3371 ions/nm^2^. (j) Overlay of an FSD image with a phase map. Blue corresponds to the Cu phase, red corresponds to the Cu_3_Ga phase. (k) IPF X orientation map. (l) Band contrast and grain boundary map. HAGB are displayed in red, LAGB are displayed in yellow. (m) KAM map, green shows higher misorientation. (n–q) 25 keV Ne, 88.5° incidence irradiation with 2247 ions/nm^2^. (n) FSD image showing the polished and unpolished sample surface. (o) IPF X orientation map showing a polished and unpolished area on the sample. (p) Band contrast and grain boundary map of a polished as well as unpolished region on the sample. HAGB are displayed in red, LAGB are displayed in yellow. (q) KAM map of a polished as well as unpolished region on the sample. A higher kernel average misorientation is shown in green.

To evaluate the effect on the EBSD measurements and to verify the Cu_3_Ga phase with an additional independent technique, different areas were irradiated with the lowest and the highest ion dose and the EBSD orientation and phase maps were recorded and evaluated. The measurements and evaluations are shown in Figure 7a–c. The figure insets show the experiments with the lowest ion dose. The 0° incidence angle milling creates a strong surface topography in both cases, as evidenced in the forward scatter diffraction image (Figure 7a). Figure 7a shows an overlay of the FSD image with the phase map. A strong surface topography was expected as different grain orientations mill at different rates. As a result of the induced strong topography, a lot of the regions cannot be indexed, returning twice the amount of zero solutions for the pattern indexing when compared to an unpolished area close by on the sample. The extent of the zero solutions can be seen in Figure 7b where the areas of zero solutions are displayed in green. This effect is not surprising and the samples are rarely polished with a 0° incidence angle for this reason. The phase map (Figure 7a) clearly shows that both the copper (blue) as well as Cu_3_Ga (red) phase are present within the irradiated area in both cases. The Cu_3_Ga phase is located at topographically higher regions as evidenced in the overlay with the FSD. These regions mill at a reduced rate and the steady-state condition is reached later, allowing for a higher Ga impurity implantation concentration. The experimental result is in good agreement with the theoretical prediction from the Monte Carlo simulation and subsequent calculations. The Cu_3_Ga phase-transformed regions are larger for the highest ion dose when compared to the lowest ion dose. This is expected as more ions were implanted in the overall sample during the polishing process for the highest ion dose when considering not a single grain but rather a multitude of grains, in which each one reaches the steady-state condition at different times. This result is in good agreement with the observed dark patch growth in the ion channeling images. The kernel average misorientation map and grain boundary map overlay (Figure 7c) shows a higher local misorientation and low-angle grain boundaries within the topographically higher regions, suggesting a higher number of defects and strain in those regions. A higher misorientation is represented by green areas in the KAM map. This result is in good agreement with the TEM measurements which also showed an increased defect density. The faster milling grains are indexed as the Cu phase. This was expected from the calculations as the maximal Ga implantation concentration is below the critical threshold required to phase transform to Cu_3_Ga. The KAM and grain boundary evaluation do not show significant strain in these regions. The maximal number of defects and implantation concentration is lower for these faster milling grains as the steady-state condition is reached earlier and less defects are created.

The experiment shows that phase transformations of Cu to Cu_3_Ga can occur within 2 to 3 min when polishing samples using a Ga FIB. The phase transformations occur in slower milling grains. The calculations suggest that a concentration of ≈36% of Ga can be found within these topographically higher regions which exceeds the required concentration threshold to form the Cu_3_Ga phase. In addition, a higher strain and defect density can be observed here suggesting significant crystal structure alterations. Faster milling grains, however, do not reach this critical concentration and phase transformations. In addition, significant crystal structure changes could not be observed in the EBSD measurements for those grains. The measurements suggest that the damage and occurring phase transformation are linked to the speed at which the steady-state condition can be reached. For a steady-state condition that is reached sufficiently quickly (fast milling grains), sample alterations can be avoided.

#### 25 keV Ne ion irradiation at 0° incidence angle

Ga as an ion species causes problems with phase transformations from copper to Cu_3_Ga. Using a different ion species holds the potential to avoid this problem and Ne, as an inert ion species, appears as a suitable candidate. To test if Ne is suitable to polish the copper sample for EBSD measurements, the copper TEM grid was irradiated using two different ion doses 3371 ions/nm^2^ and 2247 ions/nm^2^ at a 0° incidence angle. An acceleration voltage of 25 kV is a commonly used operating parameter for patterning with neon and was chosen for that reason in these experiments. To achieve 30 keV Ne ions, the extractor would have to be raised to >37.5 kV which can lead to source blunting of a newly installed source. The value of 25 keV is sufficiently close to enable a comparison with the 30 keV Ga and allows for an easy and safe operation.

The Monte Carlo simulations show that the interaction volume of 25 keV Ne ions extends beyond the EBSD signal depth (see Figure 2). A total of 532 vacancies are produced per incident Ne ion. However, a significant portion of the vacancies are created below the EBSD signal depth (Supporting Information File 5), in contrast to Ga ions. The combination of these two effects suggests that only 1/3 of the vacancies (266 vacancies per incident ion) are created within the EBSD signal depth in comparison to the 30 keV Ga ion irradiation. Only four atoms are statistically sputtered per incident 25 keV Ne ion. This is significantly less than that for Ga (ten Cu atoms are sputtered per incident ion). The combination of a lower sputtering rate and a larger interaction volume depth for Ne is expected to result in an overall higher ion implantation concentration for Ne as the steady-state condition is reached later. To determine the dose required to reach the steady-state condition, the sample was irradiated with 3371 ions/nm^2^ and a TEM lamella was prepared. The depth for a slow milling grain was measured to be 144 nm in the cross-sectional view. Considering that the interaction volume depth is 70 nm, the steady-state condition is reached at a dose of 1638 ions/nm^2^. Throughout this time, 1.64 × 10^11^ Ne ions interact with the sample and implant in the sample. This is approximately the number of Cu atoms in the EBSD depth (2.1 × 10^11^ Cu atoms).

**Figure 8:**
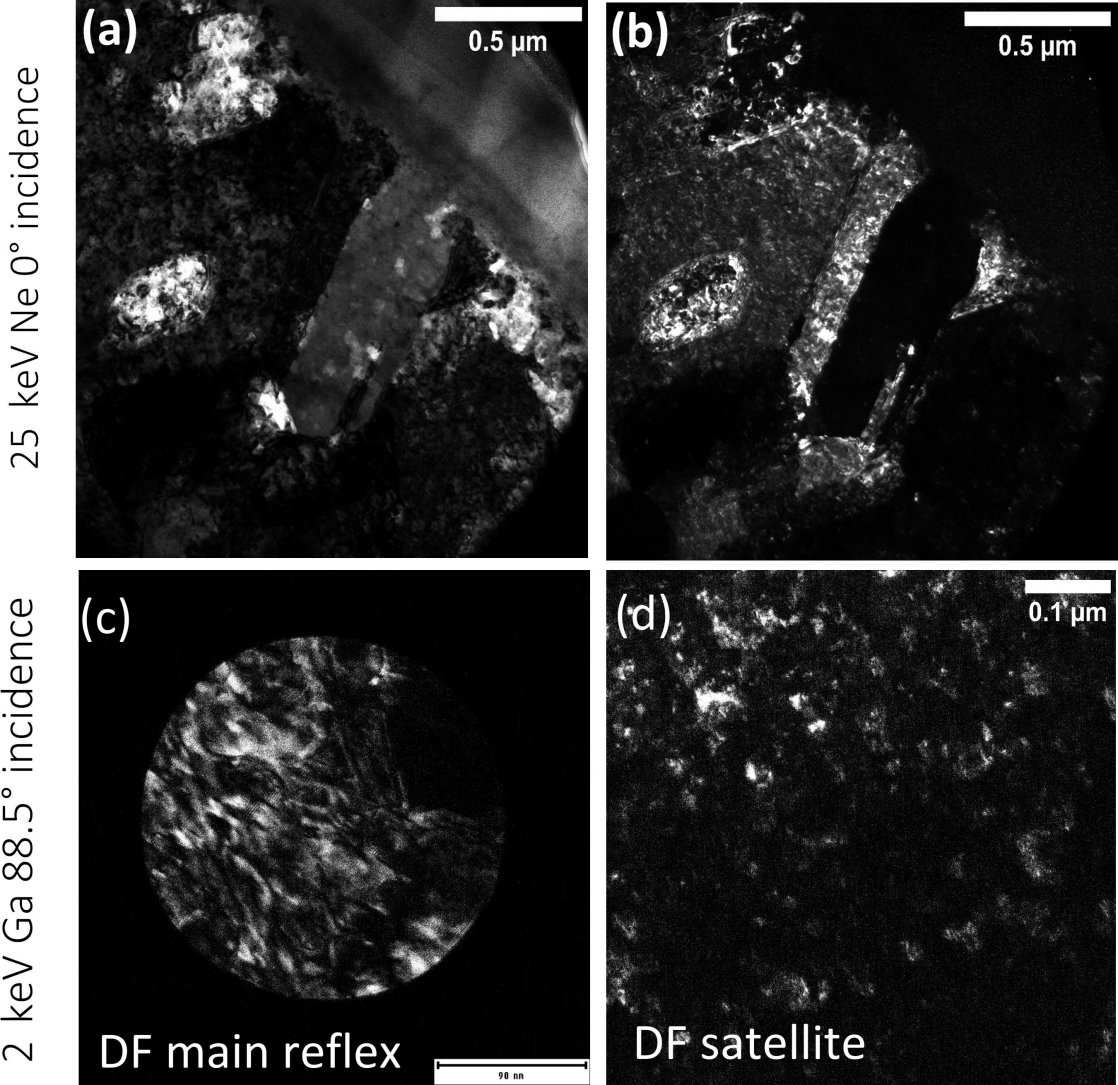
TEM measurements of a 25 keV Ne 0° incidence (top row) as well as 2 keV Ga 88.5° incidence (bottom row) irradiated Cu sample in a cross-sectional view. (a) Bright-field image of a satellite peak for the Ne-irradiated sample. (b) DF image of the satellite bright-field peak for the Ne-irradiated sample. (c) DF image of the main reflection. (d) DF image of the satellite reflection.

The TEM lamellae were prepared from both the lowest ion dose irradiated area (2247 ions/nm^2^) as well as the highest ion dose irradiated area (3371 ions/nm^2^). The TEM measurements and diffraction patterns are shown in Figure 6. The TEM measurements of the lowest dose (2247 ions/nm^2^) irradiated area, see Figure 6k, show a higher defect concentration in comparison to the control sample. A significant amount of twinning can be observed at the grain boundaries. The corresponding SAED pattern for the lowest ion dose irradiated area (2247 ions/nm^2^) is given in Figure 6l. The angles between the reflections were determined to be α = 34° and β = 71° and the distance ratio to *A*/*B* = 1.67 suggesting an fcc phase with [310] ZA. The corresponding SAED simulated pattern is shown in Figure 6m. The measured reflexes were *d*_hkl,A_ = 0.187 nm and *d*_hkl,B_ = 0.112 nm which correspond to those reported for Cu (*d*_002,Cu_ = 0.181 nm and 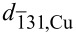 = 0.109 nm). The deviation of the *d*-spacing for the (002) reflection was also observed for the non-irradiated control experiment. The TEM measurements of the highest dose (3371 ions/nm^2^) irradiated area (see Figure 6n) show a significantly higher number of defects and line defects in comparison to the control sample. The corresponding SAED pattern is given in Figure 6o. The angles between the reflections were determined to be α = 52° and β = 70° and the distance ratio to *A*/*B* = 1.15 suggesting an fcc phase with [011] ZA. The corresponding simulated SAED pattern is given in Figure 6p. The measured reflections were *d*_hkl,A_ = 0.217 nm and *d*_hkl,B_ = 0.189 nm which correspond to those reported for Cu (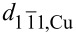 = 0.209 nm and *d*_200,Cu_ = 0.181 nm). The distances as well as the angles between the reflections show deviations from the expected literature values. For example, the (200) and the (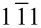) reflections show deviations of ≈0.01 nm. This behavior was previously observed for the (200) reflection only in the control sample. The enlarged *d*-spacing values for the (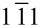) reflection suggest additional strain within the lattice. Furthermore, satellite peaks can be seen around the major reflections for this higher ion dose. To further assess the additional reflections, dark-field images were recorded for a satellite peak (see Figure 8a and Figure 8b). The bright areas in the dark-field image, which correspond to the satellite peak, are located around the grain boundaries and extend several hundreds of nanometers into the sample. A total of 4.36 × 10^13^ vacancies are created within the EBSD signal depth until the steady-state condition is reached. This suggests that every Cu atom would statistically be displaced 266 times during the irradiation process. These extremely high numbers of implanted Ne and created defects are likely the cause of the significant crystal alterations observed in the TEM measurements here. The calculations presented above concern a specific grain which was milled more slowly than other grains. According to this calculation, the steady-state condition is reached before the lowest ion dose is delivered. It would, therefore, be expected that the lowest and highest ion doses would yield the same result. The calculation, though, represents only a single measured grain and does not consider faster milling grains or grains which were milled slower. These grains would reach the steady-state condition at different stages and the delivered ion dose may make a big difference for those grains and would explain the observed results. Overall, the highest ion dose would produce more defects and a higher impurity concentration when averaged over many different grains which agrees with the TEM results. A 0° incidence irradiation with Ne produces bubbles directly underneath the sample surface (Figure 6j). The depth of the bubble layer directly corresponds to the interaction volume depth and the size of the bubbles corresponds to the distribution of ion ranges. Bubble formation is a well-documented artefact for He [41]. It is not surprising to find this for Ne considering the excessive number of implanted Ne ions resulting in oversaturation and hence bubble formation for this polishing procedure.

To evaluate the effect on the EBSD patterns, EBSD orientation maps for both the highest (3371 ion/nm^2^) and lowest (2247 ions/nm^2^) ion dose irradiated areas were recorded and evaluated. In both cases, the amount of zero solutions could be reduced by 1/3 and the patterns indexed within the polished area. The IPF X orientation map of the irradiated areas and the surrounding unpolished Cu is given in Figure 7d. A careful evaluation of the grain boundaries in both cases shows a significantly higher percentage of LAGB (see Table 1) with 66% when compared to the control sample with 8%. The evaluation was performed on EBSD measurements of individual areas to avoid contributions from the non-irradiated areas of the sample shown in Figure 7d. The band contrast and grain boundary maps for the irradiated areas are shown in Figure 7e and Figure 7g. The LAGB are displayed in yellow and the HAGB are displayed in red. The KAM maps (Figure 7f and Figure 7h) show a significantly higher level of misorientation in the irradiated sample (shown in green in the map). The measurements suggest changes in the microstructure and this is in good agreement with the TEM measurements which suggested a higher defect density and presented satellite peaks in the SAED pattern for the highest ion dose irradiation. Even though phase transformations could not be observed, significant crystal structure alterations as well as Ne bubble formation throughout the EBSD signal depth occurred when processing the sample at a 0° incidence angle, suggesting that this setting is unsuitable and can lead to EBSD result misinterpretation, especially for strain and deformation analysis.

### Glancing angle irradiation of copper

Polishing samples at glancing angles with Ga FIBs using low energies, often for prolonged periods of time, is commonly reported [42–43]. The polishing performance of Ga FIB as well as of HIM operated with Ne was tested for glancing angle irradiation. The different irradiation measurements are illustrated in Figure 2.

#### 2 keV Ga ion irradiation at a glancing angle

The Monte Carlo simulations (Figure 2) show that the interaction volume of the 2 keV Ga ions is reduced to the top of the EBSD signal information depth with ≈3 nm when using glancing angles. This suggests that a significant portion of the sample should be unaltered in the EBSD signal volume.

**Figure 9:**
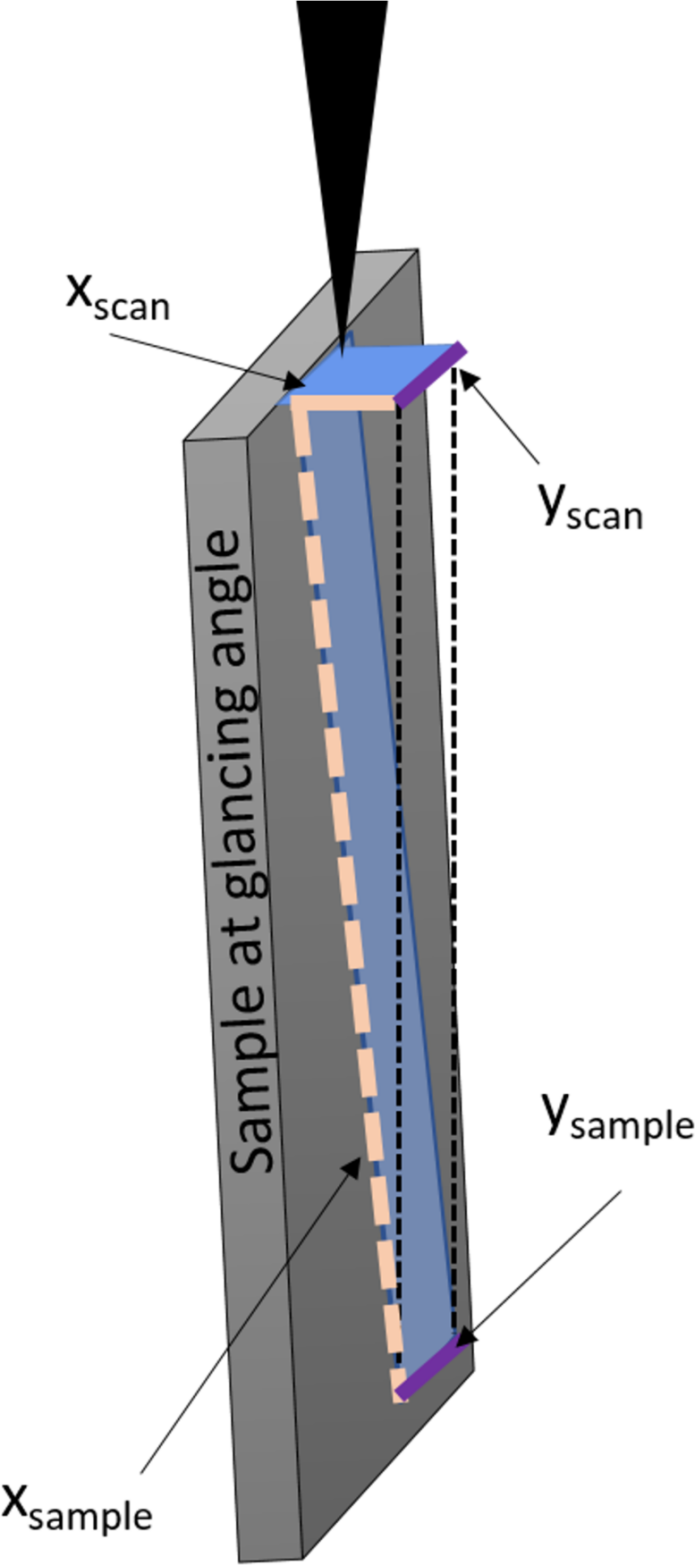
Illustration showing the enlarged irradiation area resulting from polishing at glancing angles. The scan size remains unchanged in the *y*-direction which is indicated by purple lines. The irradiated area is increased in size in the *x*-direction as a result of the projection of the setup irradiation area onto the tilted sample surface (light orange lines).

As phase transformations started to occur for the lowest ion dose, the lowest dose of 2247 ions/nm^2^ was used here. To irradiate a 100 µm^2^ area with 2247 ions/nm^2^, a longer irradiation time is required for the glancing angles in comparison to a 0° incidence angle irradiation. This can be understood when considering the projection of the irradiation area on the actual sample area. The area on the sample itself is larger as a result of the glancing angle which is illustrated in Figure 9. The total irradiation time of 33 min was used and was determined in a pre-experiment in which the polished area on the sample was measured. This approach was chosen, rather than determining the required time via calculation, since small deviations in the glancing angle have a large effect on the actual irradiated area and, therefore, on the required dose. For example, a glancing angle of 86.5° would result in a 1650 μm^2^ area while an 88.5° angle would result in a 3820 μm^2^ area. The TEM copper grids can be slightly tilted in a holder and since such small angular changes have significant effects, a more accurate pre-experimental verification was used to determine the time required for delivering 2247 ions/nm^2^. Polishing times on the order of 30 min at glancing angles are often reported in the literature and, therefore, represent a meaningful parameter. The height difference was measured to be 26 nm in TEM images for slower milling grains which means that the steady-state condition is reached after 228 s. Throughout this time, 4.27 × 10^11^ Ga ions interact with the sample and this represents the upper limit for Ga impurity concentration in the sample. There are only 4.19 × 10^11^ Cu atoms within the volume defined by the irradiated area (1650 μm^2^) and the interaction volume depth is 3 nm. While the majority of the sample should remain unaltered, there is, in theory, an excessively high Ga impurity concentration in the top layer which is above the Ga concentration threshold required to form a Cu_3_Ga phase. The Monte Carlo simulations suggest that 22 vacancies are created per incident ion. As a result, 9.4 × 10^12^ vacancies are created within the first 3 nm until the steady-state condition is reached. This number is larger than the actual number of Cu atoms, suggesting that every Cu atom is dislocated approximately 22 times from a lattice position. The calculation suggests that the Cu atoms are drastically moving throughout the irradiation process but are less often displaced in comparison to 30 keV Ga (see Table 1). The sputtering rate is significantly reduced with five Cu atoms per incident ion in comparison to the frontal irradiation with 30 keV Ga ions (see Table 1). The resulting slower milling leads to an increased time until the steady-state condition is reached and, therefore, higher defect density and impurity concentration are expected.

Throughout the experimental ion irradiation process, changes in the ion image contrast occur with darker stripes forming within the grains in the irradiated area (see Figure 5d). They cover 24% of the irradiated area. This value was determined using a threshold analysis in the image analysis program FIJI. The measured width of the stripes ranges from 70 to 150 nm. The corresponding FSD measurement (Figure 7j) shows that these stripes are closely related to differences in the sample topography.

A TEM lamella was prepared from the top part of the irradiated area and diffraction patterns underneath the evaluated sample surface. The TEM image (see Figure 6r), recorded directly underneath the sample surface shows a significant number of defects and precipitates. The SAED pattern (Figure 6s) shows a distinctly different behavior of both the control experiment and the 30 keV Ga 0° incidence angle irradiation experiment, with satellite reflections surrounding the main reflection. A similar diffraction pattern was previously observed for the periodic antiphase structure of CuAuII [42]. It is unlikely that the reflections are caused by oxide growth on the free Cu surface. If the satellite reflexes were caused by double diffraction (first from the Cu layer and then from the oxide layer) the satellite peaks would be observed for all lamellae as they were all prepared equal. The satellite peaks would also be present on both the irradiated as well as the non-irradiated area of the TEM lamella. For TEM lamella preparation, the irradiated area was immediately covered with a protective layer in situ, making oxidation unlikely. The angles between the reflections were measured to be α = 55° and β = 62° and the ratio of distances determined to be *A*/*B* = 1.05 for the main reflection. The pattern matches best a hcp structure with [011] ZA. The corresponding simulated SAED pattern is given in Figure 6t. The next best possible candidate would be a drastically distorted fcc structure with 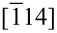 ZA. The angles of α = 65° and β = 50° as well as the ratio of *A*/*B* = 1.17 for the fcc structure are significantly different, making this an unlikely candidate. A comparison between the reported *d*-spacing values for Cu (*d*_hkl,A_ = 0.128 nm and *d*_hkl,A_ = 0.109 nm) and the measured *d*-spacing values *d*_hkl,A_ = 0.220 nm and *d*_hkl,B_ = 0.201 nm shows a significant mismatch, suggesting that the SAED pattern does not correspond to a Cu fcc structure with a 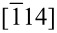 ZA. Instead, the pattern matches the diffraction pattern for the Cu_3_Ga phase with a [011] ZA. The measured *d*-spacing values match the reported *d*-spacing values *d*_100_ = 0.225 nm and 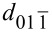 = 0.200 nm for the Cu_3_Ga hcp structure. To further evaluate the satellite peaks, dark-field images were recorded for the main reflection peak and the satellite peak. The corresponding measurements are shown in Figure 8c and Figure 8d. The image shows that small clusters light up in the dark-field image of the satellite peak which have similar sizes as the observed stripes in the ion channeling image in Figure 6r. Anti-phase domains, which have been reported to produce such satellite spots in intermetallics such as CuAuII [44] can be understood as planar crystallographic defects. In anti-phase domain regions, every atom sits on its anti-site which can be imagined as a region consisting of anti-site defects. These regions could have been formed as a result of the high Ga implantation concentration and induced dislocations/vacancies within this area. The same behavior was observed for the 25 keV Ne 0° incidence angle irradiation for which an equally high number of implanted ions and defect formation was observed.

The TEM measurements show that defects and precipitates occur predominantly around grain boundaries and that these features can be found at a significant distance below the sample surface. The evaluation of the SRIM simulation suggested that each copper atom is displaced around 22 times during the polishing process. A high enough Cu atom as well as Ga ion mobility is likely to move ions as well as displaced Cu atoms into grain boundaries. Grain boundaries can facilitate transport deeper into the material and this could significantly alter the sample below the initially expected 3 nm alteration layer and would explain the position of defects/inclusions in the TEM images and bright clusters in the dark-field image. To assess this theory, EDS measurements were performed on the TEM lamella using 30 keV electrons. An overlay of a dark-field STEM image and the background-subtracted, peak-deconvoluted, 1σ-thresholded EDS Ga map (Tru map) is shown in Figure 6q. The different grains are clearly visible in the DF STEM image. The overlay shows that the Ga (black spheres) can end up several hundreds of nanometers below the sample surface along the grain boundaries. A sufficiently high Ga concentration can be reached in some areas and a phase transformation can occur much deeper within the sample than initially anticipated with 3 nm. The measurement shows that the initial assumption of a limited damage layer of 3 nm is incorrect. However, those sample alterations can occur throughout the entire EBSD signal depth (and beyond) once the Ga migrates along the grain boundaries.

To evaluate the effect on the EBSD pattern, an orientation and phase map of an irradiated area was recorded. Less surface topography was induced in comparison to a 0° incidence irradiation which allows better indexing and a significantly lower zero solution return (zero solution by ≈66% in comparison to the surrounding area). The overlay of the FSD image with the phase map (Figure 7j) shows that the Cu_3_Ga phase can be found within the topographically higher regions. The same behavior was also observed for the frontal irradiation with 30 keV ions suggesting that 30 min polishing with Ga ions at glancing angles can induce phase transformations on copper in slower milling grains. This is in good agreement with the initial calculations suggesting that a sufficiently high Ga impurity concentration is reached within the top layer of the EBSD signal depth during the irradiation process for those grains. The phase-transformed areas are significantly smaller in comparison to the 30 keV Ga frontal polishing. A reason as to why this is could be the differences in the final sample topography after polishing. The resulting topographically higher regions are significantly smaller in area when polishing at glancing angles with 2 keV Ga, as evidenced in the FSD image (Figure 7j). This limits the area size in which a phase transformation to Cu_3_Ga can occur. This effect would explain why the overall phase-transformed area is significantly smaller here despite a resulting higher Ga impurity concentration in comparison to the 30 keV Ga frontal polishing. It is plausible that the excess of Ga ions are incorporated into the lattice once the required impurity concentration for a phase transformation to Cu_3_Ga is locally reached. The evaluation of the grain boundaries shows distinct deviations from the control sample. A significantly higher amount of LAGB is now present in the sample (see Figure 7l), with 17% in comparison to 8% observed for the control sample. The percentage of the different grain boundaries can be found in Table 1. LAGB can be indicators of recrystallization processes and can be observed for materials with higher defect densities. The KAM map (Figure 7m) shows a higher level of misorientation in the irradiated sample (shown in green in the map). This is in good agreement with the TEM measurements. It is not surprising to see alterations to the crystal structure as a result of the ion–solid interactions, especially considering the resulting impurity concentration and number of created defects. While a well-indexed EBSD map can be obtained using this polishing technique, significant crystal structure alterations as well as localized phase transformations occur making this approach unsuitable for polishing Cu samples without inducing significant sample alterations.

#### 25 keV Ne ion irradiation at 88.5° glancing angle

Since EBSD polishing is conventionally done at glancing angles, the Cu sample was irradiated with Ne at a glancing angle. Ne ions at 25 keV were used for these experiments. This setting was chosen over a lower ion energy as the interaction volume depth for both 25 keV Ne ions as well as 10 keV Ne ions (lowest possible ion landing energy) extends beyond the EBSD signal depth. Furthermore, the extractor voltage would need to be lowered to achieve sub-10 keV ion energies which significantly compromises Ne stability during the time-consuming polishing process and which should be avoided to ensure an even polishing. Furthermore, the sputtering yield for 10 keV Ne ions at an 88.5° glancing angle incidence is eight and thus smaller than that for 25 keV Ne ions. This would lead to a higher required dose to reach the steady-state condition and thus has the potential to create more defects.

The Monte Carlo simulations (Figure 2) show that the interaction volume of 25 keV Ne ions extends only marginally beyond the EBSD signal depth with 30 nm. The number of vacancies that are induced within the EBSD signal length is significantly reduced with ≈170 vacancies per ion while the number of sputtered atoms (12 atoms per incident ion) is the highest for the different polishing setups. The sputtering rate limits the overall number of created vacancies as well as the ion implantation concentration. A milling depth of 220 nm was achieved during the irradiation and significant topographic differences between grains were not observed. The steady-state condition is reached after 3.0 × 10^10^ Ne ions have interacted with the sample. This corresponds to a maximal impurity concentration of 12% which is significantly lower than that for a 0° incidence irradiation. Throughout this time, each Cu atom is displaced from its lattice site 17 times. Both the resulting impurity concentration as well as the number of created Cu displacements/vacancies are the lowest for this polishing experiment in comparison to the frontal Ne polishing and all Ga polishing experiments (see Table 1).

The cross-sectional view of the TEM measurement of the irradiated area is shown in Figure 6u. A significant number of defects and interstitials cannot be observed here and indeed the sample has a similar appearance of the control experiment. The SAED pattern (Figure 6v) was recorded in a position underneath the sample surface on the prepared TEM lamella. The angles were determined to be α = 52° and β = 70° and the distance ratio *A*/*B* = 1.15 suggesting an fcc phase with [101] ZA. The corresponding simulated SAED pattern is given in Figure 6w. The *d*-spacing values were measured to be *d*_hkl_ = 0.215 nm and *d*_hkl_ = 0.189 nm which are in agreement with those reported for Cu (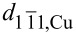 = 0.209 nm and *d*_200,Cu_ = 0.181 nm). The diffraction pattern corresponds to that of the copper phase with a [101] ZA. The deviation for the (200) reflection was also observed for the control sample and the results are overall in good agreement with the control experiment, suggesting that no significant crystal structure alteration occurred using this polishing protocol. Bubble formation could not be observed in the TEM images. The impurity concentration is likely not high enough for the required supersaturation and subsequent bubble formation. Furthermore, the reduced range of Ne ions at glancing angles would produce bubbles directly underneath the sample surface rather than at a significant distance underneath the sample surface. The closest distance to the sample surface, from which gas bubbles could escape, as well as the highest removal rate of atoms at the sample surface (due to the highest sputtering yield, see Table 1) are both likely to contribute to a lower overall amount of Ne accumulation in the sample, making bubble formation as well as interstitials less likely.

The FSD image of the sample (Figure 7n) shows the difference between the polished and unpolished sample area. The EBSD IPF X orientation map (Figure 7o) of non-polished sample areas as well as a 10 µm wide polished stripe show that a significant improvement in indexing can be achieved when polishing the sample with 25 keV Ne ions at a glancing angle. Overall, zero solutions could be halved. An evaluation of the grain boundaries (see Figure 7p) shows that 92% of the grain boundaries are high-angle grain boundaries and 8% are low-angle grain boundaries which is in good agreement with the control experiment. The KAM map (Figure 7q) shows no difference in misorientation between the sample and the polished area, suggesting that significant changes to the crystal structure were avoided.

The results suggest that Ne ions are a suitable ion species to polish copper for EBSD measurements. Phase transformations to Cu_3_Ga could be avoided by using the inert ion species. Crystal structure alterations could be avoided when polishing at high energy at glancing angles. The measurements and Monte Carlo simulations suggest that significant crystal structure alterations occur for a sufficiently high ion implantation concentration as well as defect formation within the sample. Minimizing these is essential to avoid ion-beam-induced artefacts for EBSD measurements and polishing with higher energy Ne ions (25 keV) at glancing angles achieves exactly this.

To verify if Ga milling would lead to similar significant artefacts in 3D EBSD applications when using a Ga FIB/SEM, the intersection of the irradiated Cu and the non-irradiated Cu was analyzed. TEM lamellae were prepared containing regions of both polished and unpolished sections of the sample. The altered area could only be found directly underneath the polished area and did not appear to significantly extend sideways for this material (see Supporting Information File 6) when using high-energy (30 keV) Ga ions. The ion-beam-exposed area was milled away and was not considered in EBSD measurements for 3D reconstructions. This concept is illustrated in Supporting Information File 1. This would explain why problems have not yet been reported with 3D EBSD. Considering the migration of ions along the grain boundaries, a more thorough assessment of potential sample alterations depending on the ion species should be carried out in future.

## Conclusion

The presented results show that ion beam polishing can induce artefacts which can easily lead to misinterpretation of EBSD measurements. The often-used Ga FIB polishing can induce phase transformation as well as significant changes in the crystal structure, even for polishing with lower ion energies at glancing angles as shown in this work for simple samples, such as Cu. While well-indexed EBSD maps could be achieved using electropolishing and PIPS, both methods introduced crystallographic artefacts in the sample, leading to a higher number of LAGB and a higher KAM. The results suggest that polishing with high-energy Ne ions (25 keV) at glancing angles significantly improves indexing without changing the crystal structure of the sample. A detailed study on microtextural modifications and texture development with respect to easy channeling direction as well as a function of grain size should be carried out in different materials in the future. The evaluation of the experimental results and the Monte Carlo simulations suggest that impurity concentration as well as atom displacements within the sample are the key drivers for phase change and significant crystal structure changes. Minimizing the resulting ion implantation concentration as well as the number of displacements per individual sample atom within the EBSD signal depth is essential to avoid artefacts. This is achieved by quickly reaching the steady-state condition. The results show that the high sputtering yield of the 25 keV Ne when polishing at glancing angles leads to a quickly achieved steady-state condition which limits impurity concentration as well as atom displacements within the sample. This work shows that the HIM operated with Ne is a suitable instrument for EBSD sample preparation and it is able to prepare perfect samples for EBSD investigations of Cu.

## Experimental

### Control experiment

To study the native crystal structure of the sample, EBSD measurements were performed on a conventional bulk copper TEM grid (Omniprobe Cu TEM grids) which had not been irradiated by ions.

### Irradiation of copper

Conventional copper TEM grids (Omniprobe Cu TEM grids) were irradiated using different ion species (Ga and Ne), different incident ion energies (30, 25, and 2 keV), different doses (3371 and 2247 ions/nm^2^), as well as different incidence angles (0° incidence and glancing angle irradiation). The irradiation experiments were performed using the Zeiss Orion Nanofab Helium Ion Microscope (Ne) as well as the Thermo Fisher, Scios (Ga) FIB/SEM.

### Lamella preparation using FIB and TEM

The TEM lamellae were prepared using the Thermo Fischer Scios Ga FIB/SEM. The irradiated area was identified by using SEM (30 kV acceleration voltage, 25 pA) and a lamella was prepared such that half of the TEM lamella had an irradiated area while the other half of the lamella had a non-irradiated area for the control measurement. This approach was chosen to ensure that the TEM lamella preparation did not alter the irradiation area in any way and that all regions surrounding the irradiated area were equivalent on the sample. A >500 nm thick platinum layer was deposited using the electron beam (2 keV, 2.3 nA) to protect the sample surface from any potential ion beam alteration throughout the subsequent processes, including the ion–solid interactions which occur with the sample during the first few moments of the ion beam deposition that could alter the sample surface. A further 1 μm thick platinum layer (16 μm long, 2 μm wide) was then deposited using the ion beam (30 kV acceleration voltage, 0.3 nA beam current). The cross-sections were cut using a 30 kV acceleration voltage and 7 nA beam current. The region of interest was then cut out and lifted onto a TEM grid where it was subsequently thinned using 0.5 nA and 0.1 nA beam current at a stage tilt of ±1.8° from the perpendicular ion beam incidence until the electron transparency was reached for 5 keV electrons (SEM). The lamella was then polished with the ion beam using a 5 kV acceleration voltage and 16 pA beam current and subsequently with 2 kV acceleration voltage and 27 pA beam current at a stage tilt of ±6° from the perpendicular ion beam incidence. The 2 kV polish was performed to reduce the damage layer on each side of the TEM lamella.

### Alternative TEM lamella preparation: argon ion milling

To ensure that the FIB TEM lamella preparation did not alter the crystal structure for the TEM measurements, additional thin foils were prepared using argon ion mill, which is considered a conventional TEM sample preparation technique. For the argon ion milling, a Gatan PIPS was used. The conventional copper TEM grids (Omniprobe Cu TEM grids) were thinned down using the following steps: 4 keV, 5° top, 3° bottom, 3 rpm for 30 min followed by 2.5 keV, 4° top, 2° bottom, 3 rpm for 5 min and subsequently 0.5 keV, 5° top, 3° bottom, 3 rpm for 3 min.

### Electropolishing

Conventional copper TEM grids (Omniprobe Cu TEM grids) were electropolished using a Fishione Instruments Model 120 twin-jet polisher. An 82 wt % of phosphoric acid bath was used for the experiments. The samples were polished using 1–8 V DC using the speed 4 polish 1 settings. The recipe for copper samples was provided in the user manual of the system. The sample was polished for 10 s for EBSD measurements. An additional sample was electropolished for 15 s to electron transparency to create a control sample for the TEM measurement.

### Transmission electron microscopy

All TEM measurements were performed using a JEOL 2100 TEM. Electron diffraction measurements were performed using alpha 2, spot 4, condenser aperture 4, and no objective aperture. The TEM imaging was performed using alpha 2 spot 1, condenser aperture 4, and objective aperture 1. Centered dark-field imaging was performed using alpha 2 spot 1, condenser aperture 4, and a sufficiently small objective aperture which allowed to select the satellite peaks.

### Scanning transmission electron microscopy

All STEM measurements were performed inside the Thermo Fischer Scios Ga FIB/SEM using the STEM detector with exception of the STEM/EDS combined measurement which was performed using the Tescan S8000X Xe plasma FIB/SEM. In both cases a 30 kV acceleration voltage was used.

### Electron backscatter diffraction

All EBSD measurements were performed on a bulk Cu TEM grid specimen to ensure that the sample was not altered or processed in any way other than by the ion irradiation itself. The EBSD measurements were performed using an Oxford Symmetry EBSD detector on a Tescan S8000X Xe plasma FIB/SEM. The EBSD maps were recorded using the following SEM parameters: 20 kV, 300 pA, 6 mm WD, and 70° pre-tilted sample holder.

The following EBSD detector parameters were used for all EBSD experiments: resolution mode, 50 ms exposure time, solver settings: optimized TKD solver and 10 Hough bands. For larger area maps (non-polished sample, all Ne-irradiated samples) with a 39 μm field of view, a 39 nm step size was used. In addition, maps with a field of view of ≈15 μm were recorded for the statistical evaluation of all irradiated areas. A 15 nm step size was used for these measurements.

### Energy-dispersive X-ray spectroscopy

The EDS point spectra were recorded using an Oxford Ultim Max 100 EDS detector on the Tescan S8000X Xe plasma FIB/SEM. The EDS maps were background subtracted and peak deconvoluted (TruMap). In addition, a 1σ threshold was applied to ensure that only statistically significant data was included in the maps. The SEM parameters (30 kV acceleration voltage and 1 nA beam current) were chosen in the analytical mode.

## Supporting Information

File 1 STEM image showing a cross-sectional view of an ion-polished Cu sample (30 keV Ga, 3371 ions/nm^2^). Differences in the milling depth for different grains are visible.

File 2 Ion channeling video showing a Cu sample changing during Ga ion polishing.

File 3 Energy-dispersive X-ray point spectra recorded on a slower milling grain and on a faster milling grain of a 30 keV Ga ion-polished Cu sample. A dose of 3371 ions/nm^2^ was used for the polishing experiment.

File 4 SRIM simulation showing vacancies for 25 keV Ne ion irradiation of Cu at a 0° incidence angle.

File 5 STEM image showing the interface of a non-irradiated area and a 30 kV Ga 0° incidence irradiated area.

File 6 SAED patterns of a non-irradiated Cu sample prepared by electropolishing, PIPS, and FIB. In all cases, the SAED pattern matches that of Cu. (a) Electropolishing, the SAED pattern matches the [110] ZA for Cu. The measured *d*-spacing for *d*_200,A_ = 0.177 nm is slightly smaller than the reported value of *d*_002,Cu_ = 0.181 nm for Cu (PDF number 00-004-0836); however, within an acceptable discrepancy for this *d*-spacing. The measured *d*-spacing for *d*_11−1,B_ = 0.209 nm matches the reported value of *d*_111,Cu_ = 0.209 nm. (b) PIPS, the SAED pattern matches the [310] ZA for Cu. The measured *d*-spacing for *d*_131,A_ = 0.209 nm matches the reported value of *d*_111,Cu_ = 0.209 nm. The measured *d*-spacing for *d*_200,B_ = 0.189 nm is slightly larger than the reported value of *d*_002,Cu_ = 0.181 nm for Cu (PDF number 00-004-0836); however, within an acceptable discrepancy for this *d*-spacing. (c) FIB-prepared TEM lamella, the SAED pattern matches the [100] ZA for Cu. The measured *d*-spacing for *d*_022,A_ = 0.133 nm is in good agreement with the reported value of *d*_022,Cu_ = 0.128 nm for Cu (PDF number 00-004-0836). The measured *d*-spacing for *d*_002,B_ = 0.192 nm is slightly larger than the reported value of *d*_002,Cu_ = 0.181 nm for Cu (PDF number 00-004-0836), however; within an acceptable discrepancy for this *d*-spacing. The SAED measurements recorded on all the TEM lamellae which were prepared using different methods are in good agreement, suggesting that the FIB TEM lamella preparation did not significantly alter the TEM measurement results.
