# Supplementary figures and images for: Is the Ne operation of the helium ion microscope suitable for electron backscatter diffraction sample preparation?
Source: Beilstein J Nanotechnol. 2021 Aug 31;12:965–83. doi: 10.3762/bjnano.12.73 (PMC8450971; doi:10.3762/bjnano.12.73)

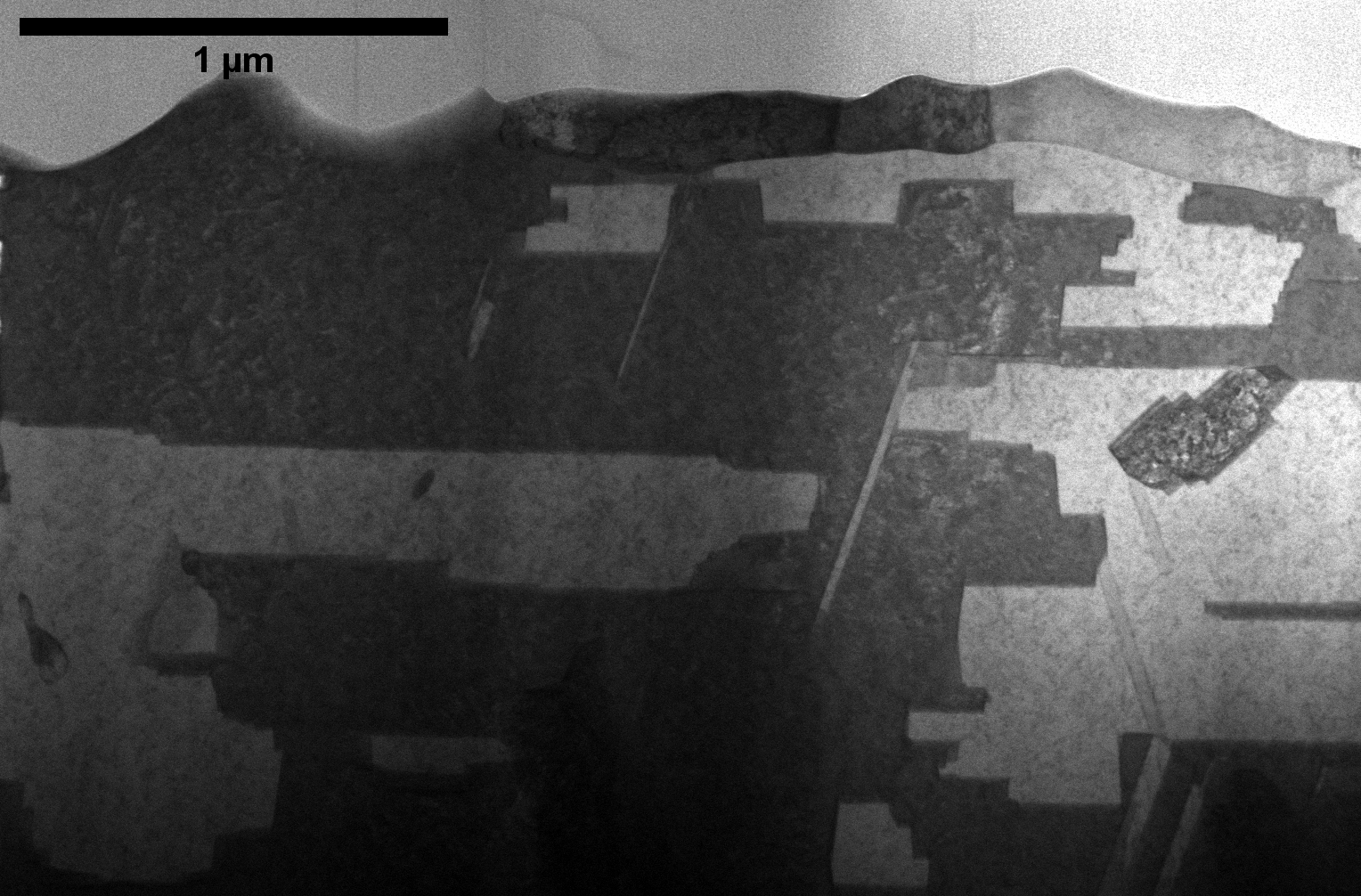

Supplement: File 1 — STEM image showing a cross-sectional view of an ion-polished Cu sample (30 keV Ga, 3371 ions/nm2). Differences in the milling depth for different grains are visible. [file Beilstein_J_Nanotechnol-12-965-s001.tif]

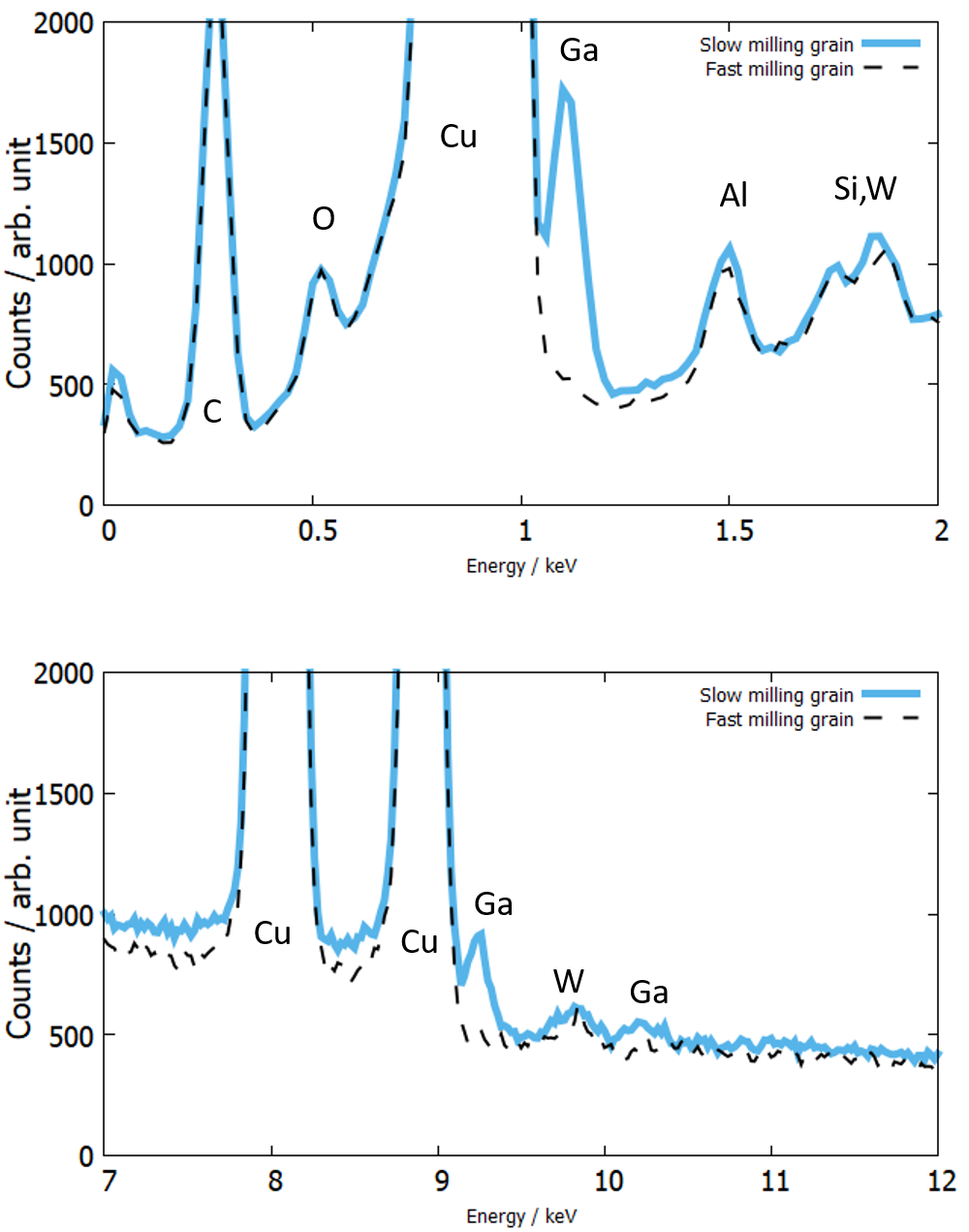

Supplement: File 3 — Energy-dispersive X-ray point spectra recorded on a slower milling grain and on a faster milling grain of a 30 keV Ga ion-polished Cu sample. A dose of 3371 ions/nm2 was used for the polishing experiment. [file Beilstein_J_Nanotechnol-12-965-s003.png]

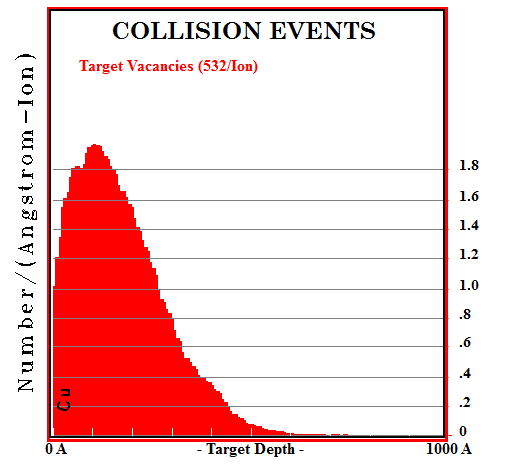

Supplement: File 4 — SRIM simulation showing vacancies for 25 keV Ne ion irradiation of Cu at a 0° incidence angle. [file Beilstein_J_Nanotechnol-12-965-s004.bmp]

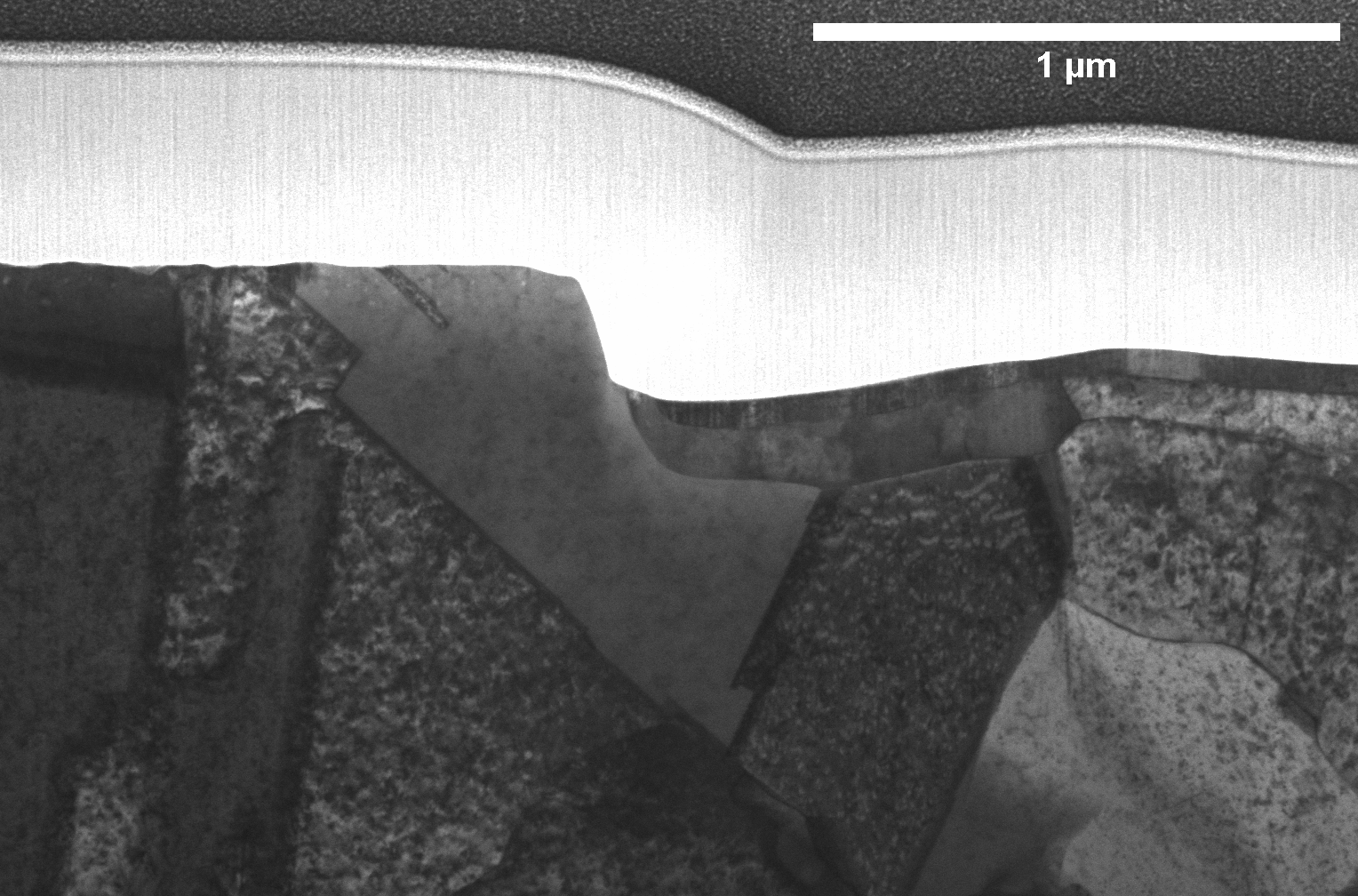

Supplement: File 5 — STEM image showing the interface of a non-irradiated area and a 30 kV Ga 0° incidence irradiated area. [file Beilstein_J_Nanotechnol-12-965-s005.tif]

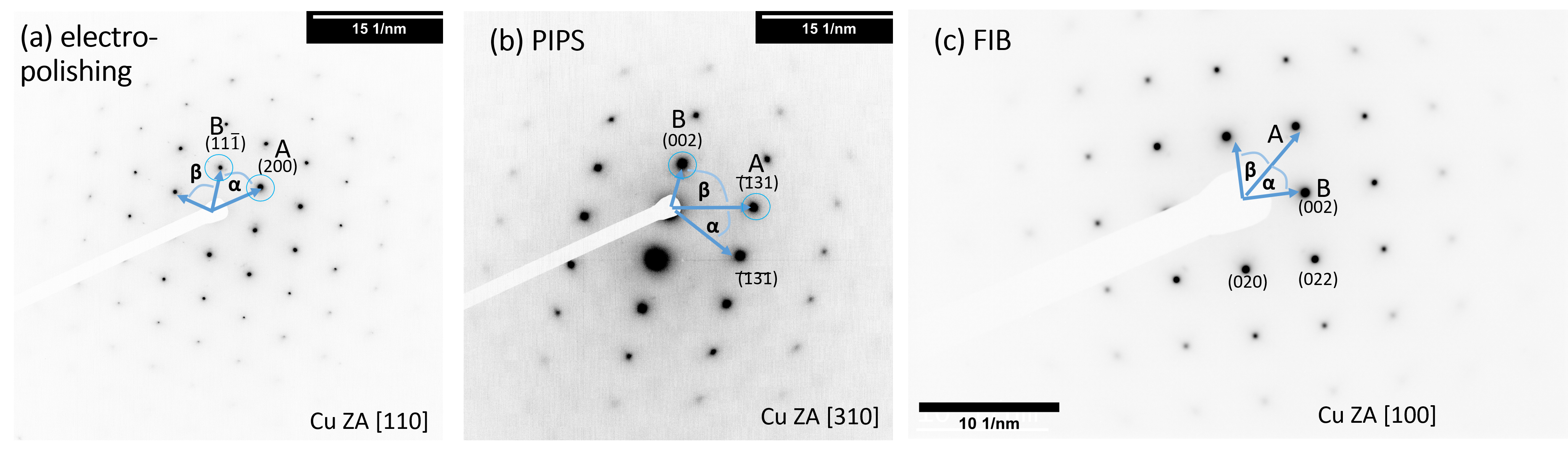

Supplement: File 6 — SAED patterns of a non-irradiated Cu sample prepared by electropolishing, PIPS, and FIB. In all cases, the SAED pattern matches that of Cu. (a) Electropolishing, the SAED pattern matches the [110] ZA for Cu. The measured d-spacing for d200,A = 0.177 nm is slightly smaller than the reported value of d002,Cu = 0.181 nm for Cu (PDF number 00-004-0836); however, within an acceptable discrepancy for this d-spacing. The measured d-spacing for d11−1,B = 0.209 nm matches the reported value of d111,Cu = 0.209 nm. (b) PIPS, the SAED pattern matches the [310] ZA for Cu. The measured d-spacing for d131,A = 0.209 nm matches the reported value of d111,Cu = 0.209 nm. The measured d-spacing for d200,B = 0.189 nm is slightly larger than the reported value of d002,Cu = 0.181 nm for Cu (PDF number 00-004-0836); however, within an acceptable discrepancy for this d-spacing. (c) FIB-prepared TEM lamella, the SAED pattern matches the [100] ZA for Cu. The measured d-spacing for d022,A = 0.133 nm is in good agreement with the reported value of d022,Cu = 0.128 nm for Cu (PDF number 00-004-0836). The measured d-spacing for d002,B = 0.192 nm is slightly larger than the reported value of d002,Cu = 0.181 nm for Cu (PDF number 00-004-0836), however; within an acceptable discrepancy for this d-spacing. The SAED measurements recorded on all the TEM lamellae which were prepared using different methods are in good agreement, suggesting that the FIB TEM lamella preparation did not significantly alter the TEM measurement results. [file Beilstein_J_Nanotechnol-12-965-s006.tif]
